# Effects of poor sleep on the immune cell landscape as assessed by single-cell analysis

**DOI:** 10.1038/s42003-021-02859-8

**Published:** 2021-11-25

**Authors:** Xiuxing Liu, Binyao Chen, Zhaohao Huang, Runping Duan, He Li, Lihui Xie, Rong Wang, Zhaohuai Li, Yuehan Gao, Yingfeng Zheng, Wenru Su

**Affiliations:** State Key Laboratory of Ophthalmology, Zhongshan Ophthalmic Center, Sun Yat-Sen University, Guangdong Provincial Key Laboratory of Ophthalmology and Visual Science, Guangzhou, 510060 China

**Keywords:** Risk factors, Autoimmunity, Inflammation

## Abstract

Poor sleep has become an important public health issue. With loss of sleep durations, poor sleep has been linked to the increased risks for diseases. Here we employed mass cytometry and single-cell RNA sequencing to obtain a comprehensive human immune cells landscape in the context of poor sleep, which was analyzed in the context of subset composition, gene signatures, enriched pathways, transcriptional regulatory networks, and intercellular interactions. Participants subjected to staying up had increased T and plasma cell frequency, along with upregulated autoimmune-related markers and pathways in CD4^+^ T and B cells. Additionally, staying up reduced the differentiation and immune activity of cytotoxic cells, indicative of a predisposition to infection and tumor development. Finally, staying up influenced myeloid subsets distribution and induced inflammation development and cellular senescence. These findings could potentially give high-dimensional and advanced insights for understanding the cellular and molecular mechanisms of pathologic conditions related to poor sleep.

## Abstract (web summary)

Liu et al. use mass cytometry and single-cell RNA sequencing of healthy human blood samples to obtain a comprehensive human immune cell landscape when sleep duration is reduced. Their findings could provide highdimensional insight into the cellular and molecular mechanisms of pathologic conditions affect by poor sleep.

## Introduction

Sleep is an indispensable attribute of life, sustaining homeostasis and safeguarding against various pathological conditions^1^. In recent years, a shortened duration and worse quality of sleep among populations have confronted us with the detrimental effects of poor sleep on health and diseases^2^. In addition, the poor sleep experiences, including poor sleep quality and acute sleep loss (SL) after staying up (SU), are common among collegiate athletes^3^. In humans, poor sleep has been reported to associate with higher incidences of autoimmune diseases, tumors and infections^4–6^. Short-term sleep deficiency can impair the innate and adaptive immune systems, resulting in increased infection susceptibility and reduced vaccination effectiveness^7,8^. In addition, prolonged habitual sleep deficiency or SU all night to work can lead to chronic, systemic, low-grade inflammation and is associated with various inflammation-related diseases, such as diabetes, atherosclerosis, and neurodegeneration^9–11^. Overall, poor sleep broadly influences the immune system and links to higher diseases susceptibility.

Previous studies have explored the impact of SL on the immune system^12^. Mice suffering from SL have elevated pro-inflammatory cytokine levels, NF-κB pathway activation, and exacerbated colonic mucosal injury^13^. In addition, sleep restriction promotes brain inflammation and neural activity impairment in mice^14^. In humans, flow cytometry demonstrated increased levels of total lymphocytes as well as T cells (TCs) and B cells (BCs) in the blood after SL^15^. Moreover, whole genome microarrays identified that SL enhanced immune activation, like BCs activation, NF-κB signaling activation^16^. However, what we know about immune cells is primarily based on flow cytometric analysis, relying on previously described markers for pooled cell populations. In addition, traditional sequencing methods covered the characteristics of different cell populations and couldn’t analyze the diversity and state heterogeneity of immune cells. The unbiased high-throughput single-cell technologies provide unique opportunities to uncover gene expression and gain insights into the molecular mechanisms associated with diseases. In previous studies, we have used single-cell techniques to construct a series of immune atlas of aging and sex, expanding our understanding of the mechanisms of aging- or sex-associated diseases^17,18^. However, the single-cell alterations through which SU and SL rewire the immune system and influences susceptibility to immune diseases are poorly understood. Therefore, a comprehensive single-cell atlas of peripheral immune response influenced by poor sleep is greatly desired.

To this end, we combined mass cytometry by time of flight (CyTOF) and single-cell RNA sequencing (scRNA-seq) to analyze the properties of peripheral blood mononuclear cells (PBMCs) before and after SU all night. Overall, SU was found to contribute to a pro-inflammatory and autoreactive state of peripheral blood by reprogramming immune subset composition, gene expression signatures, enriched pathways, transcriptional regulatory networks, and cell-cell interactions. The findings provide a comprehensive atlas of the effect of poor sleep on the immune system and expand our knowledge of SU as a predisposing factor for inflammatory or autoimmune diseases and aging-related diseases.

## Results

### Study design for single-cell immunophenotyping of human blood

**Fig. 1:**
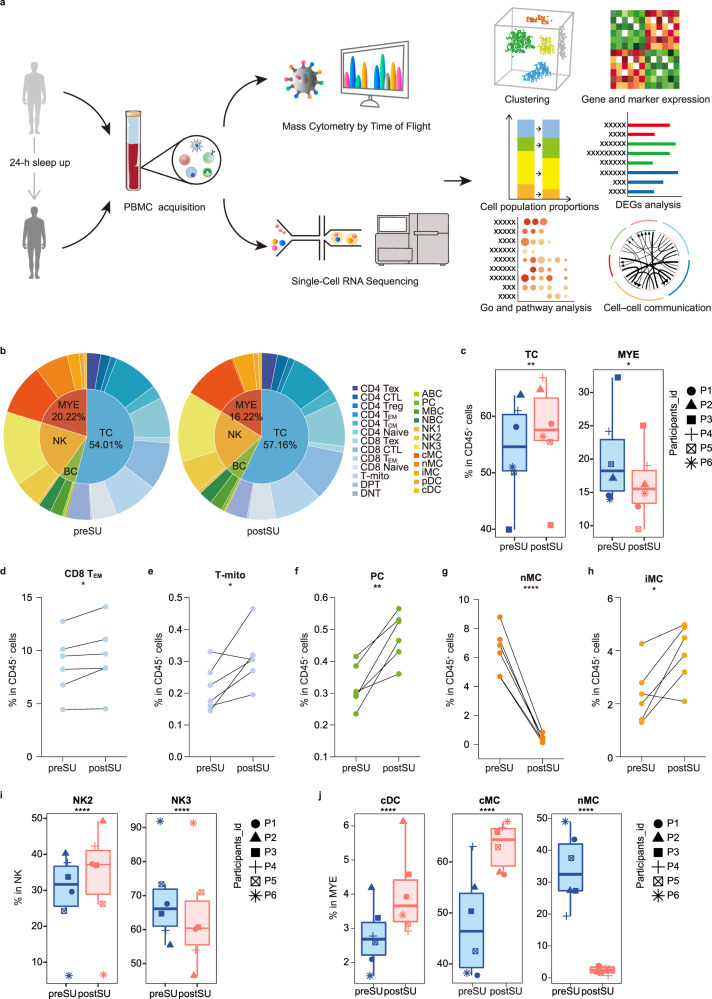
The experimental design and changes in cell proportions after SU. **a** Schematic of the experimental design for mass cytometry by time of flight (CyTOF) and single-cell RNA sequencing (scRNA-seq). Peripheral blood mononuclear cells (PBMCs) of six healthy individuals were collected before and post the 24-h sleep loss, then processed CyTOF and scRNA-seq for the subsequent studies. **b** Pie charts showing relative cluster abundance in the preSU and postSU groups. The percentage of TC and MYE (**c**), CD8 T_EM_ (**d**), T-mito (**e**), PC (**f**), nMC (**g**) and iMC (**h**) in CD45^+^ immune cell between preSU and postSU groups (*n* = 6 /group). **i** The percentage of NK2 and NK3 subsets in NK between preSU and postSU groups (*n* = 6 /group). **j** The percentage of cMC, nMC and cDC in MYE between preSU and postSU groups (*n* = 6 /group). Significance in **d**–**h** was calculated using two-tailed paired *t*-test; significance in **c**, **i** and **j** was calculated using the “diffcyt-DA-GLMM” method as implemented in the “diffcyt” function in view of the subjects pairing; *P < 0.05, **P < 0.01, ****P < 0.0001. The full names of the 25 cell types in CyTOF see Supplementary Fig. 1.

To map the human circulating immune system, identify changes in the blood, and pinpoint cell-specific alterations associated with SU, we collected blood from six healthy individuals before and after SU all night (preSU and postSU; Supplementary Table 1) and then performed CyTOF and scRNA-seq analysis (Fig. 1a). FlowSOM-defined nodes in CD45^+^ cells were manually annotated into four main immune cell types [TC, natural killer (NK) cell, BC, myeloid cell [MYE, including monocyte (MC) and dendritic cell (DC)] and then re-clustered into 25 subsets (Supplementary Figs. 1 and 2, Supplementary Table 2). Using scRNA-seq, we identified megakaryocytes (MEGA), CD34^+^ cells (CD34), and five major immune cell lineages (TC, NK, BC, MC, and DC) based on the expression of canonical lineage markers upregulated in each cluster (Supplementary Fig. 3a). We then sub-clustered five circulating immune cells into 25 transcriptionally classical subsets (Supplementary Fig. 3b–f, Supplementary Table 3).

### Reconstitution of the circulating cellular ecosystem by SU

To elucidate how cell type composition changes after SU, we compared the number and proportions of each major cell type between the preSU and postSU groups, and identified many abnormal changes (Fig. 1b, Supplementary Fig. 4a). Globally, TC frequency increased by ~3% and MYEs decreased by ~4% in postSU group (Fig. 1b, c). Next, we performed single-cell clustering to explore changes in cell subpopulations induced by SU. Following SU, we identified lymphocythemia due to an increase in the frequency of CD8^+^ effector memory TCs (CD8 T_EM_), proliferating TCs (mitotic TC, T-mito) and exhausted TCs (Tex) in CD45^+^ cells (Fig. 1d, e, Supplementary Fig. 4b). Moreover, plasma cells (PCs) were significantly upregulated (Fig. 1f). There was also a specific pattern in MC subset frequency, where non-classical MCs (nMC) were decreased and intermediate MCs (iMC) were increased (Fig. 1g, h).

Next, we explored subset composition across the corresponding cell lineages. We compared the absolute number of T-cell subsets between the two groups to identify the effects of SU (Supplementary Fig. 4c). We found that T-mito was increased and CD4^+^CD8^+^ double-positive T cell (DPT) was decreased. CD4^+^ and CD8^+^ TC subsets were similar among groups (Supplementary Fig. 4c). Analysis of number and proportion of NK clusters revealed altered subset composition, with increased NK2 and decreased NK3 (Fig. 1i, Supplementary Fig. 4d). For BCs, the number and percentage of PCs increased (Supplementary Fig. 4e, f). In MYEs, both conventional and plasmacytoid DCs (cDCs and pDCs, respectively) were increased (Fig. 1j, Supplementary Fig. 4g). Moreover, there was increased heterogeneity in the alterations of MC subset population after SU. Classical MCs (cMCs) and iMCs also increased while nMCs decreased postSU compared with preSU (Fig. 1j, Supplementary Fig. 4h).

Altogether, the CyTOF analysis results revealed complex cell dynamics in the circulation and further support the notion that this abnormal activity destabilizes blood immune homeostasis.

### Alteration of gene expression changes across subjects after SU

**Fig. 2:**
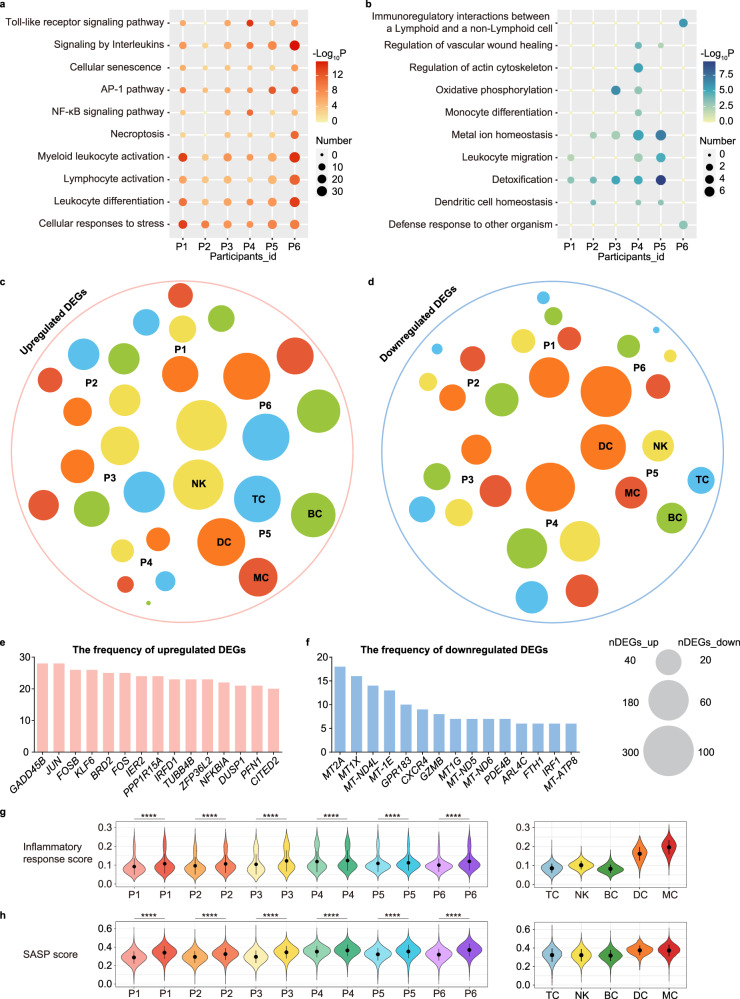
Changes in transcriptional profiles among subjects. Representative GO biological process and pathways enriched in upregulated (**a**) and downregulated (**b**) DEGs based on functional enrichment analysis among subjects. Dot plots showing the distribution of upregulated (**c**) and downregulated DEGs (**d**) in each major cell type of all six participants, the size indicates the numbers of DEGs, and the different colors annotate different cell type. Bar plots showing the frequencies of the top 15 upregulated (**e**) and downregulated (**f**) genes observed across all cell types in six participants. Violin plot of inflammatory response score (**g**) and SASP score (**h**) for each sample and immune cell lineage, different participants were represented in different colors, with darker colors representing after SU. For the box plot within each violin plot, middle lines indicate median values, boxes range from the 25th to 75th percentiles. Significance in **g** and **h** was calculated using two-sided Wilcoxon test as implemented in the function “compare_means” with default parameters; ****P < 0.0001.

To identify the molecular events associated with SU, we separated the effects of SU on each individual in this study. We generated an UpSet diagram of differentially expressed genes (DEGs) from blood immune cells in the postSU group compared with the preSU group, and found that all six participants showed an increase in some inflammatory genes, including AP-1 family genes (*JUN*, *FOS*), DNA damage markers (*DDIT3*, *GADD45B*), *IFNG*, and interferon-related developmental regulator 1 (*IFRD1*) (Supplementary Fig. 4i, j). We next explored the biological implications of upregulated and downregulated DEGs through gene ontology (GO) and pathway analysis for each subject. The commonly upregulated genes across participants after SU were enriched in AP-1 pathway, leukocyte activation, and cellular responses to stress (Fig. 2a). Notably, SU enhanced the activation of cellular senescence. SU also led to the downregulation of multiple pathways involved in metal ion homeostasis and detoxification (Fig. 2b). We next sought to define the cell type specificity of these DEGs across individuals. As indicated by the varying circle sizes, the effects of SU were not only subject-specific but also cell type-specific (Fig. 2c, d). Globally, TCs, BCs, and DCs were the cell types most strongly affected by SU among individuals according to their upregulated DEG number. Notably, *GADD45B*, *JUN*, and *FOSB* were the top three upregulated DEGs across all five major immune lineages of the six subjects after SU (Fig. 2e, f).

The increase in inflammatory pathway activity and specific gene expression after SU demonstrate that SU induces general oxidative stress and an inflammatory state. To assess the extent of these phenomena that are enhanced by SU, we calculated the score for each subject. We found that all participants exhibited a upregulation in the inflammatory response score after SU, with MYEs showing the highest inflammatory response score (Fig. 2g). In addition, we also found that in all subjects there was an upregulation of reactive oxygen species (ROS) and senescence-associated secretory phenotype (SASP) scores in the postSU group compared with the preSU group (Supplementary Fig. 4k, Fig. 2h).

### SU results in autoimmune-associated changes in effector lymphocytes

**Fig. 3:**
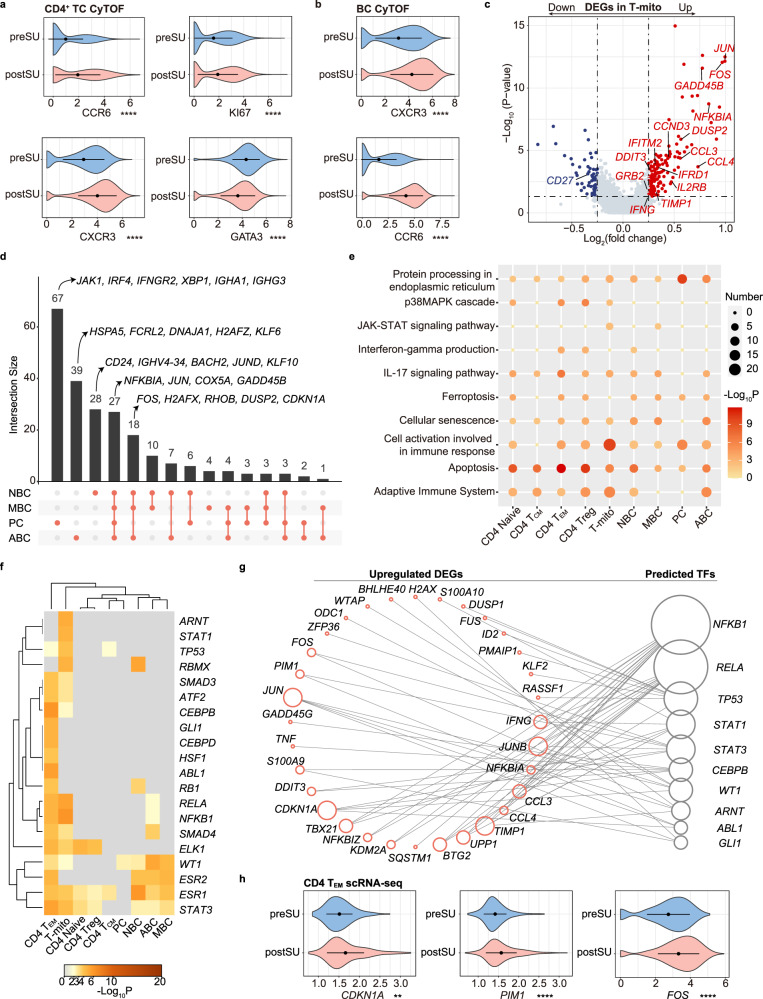
Changes in proteomic and transcriptional profiles of CD4^+^ TC, T-mito and BC. **a** Violin plot showing the expression of CCR6, CXCR3, KI67, GATA3 in CD4^+^ TC between preSU and postSU groups in CyTOF. **b** Violin plot showing the expression of CXCR3, CCR6 in BC between preSU and postSU groups in CyTOF. **c** Volcano plot showing DEGs of T-mito between preSU and postSU groups. **d** UpSet Plot showing the integrated comparative analysis of upregulated DEGs in BC subsets. **e** Representative GO biological process and pathways enriched in upregulated DEGs based on functional enrichment analysis in CD4^+^ TC, T-mito and BC subsets. **f** The heatmap showing the enhanced activity of TFs predicted by TRRUST analysis in CD4^+^ TC, T-mito and BC subsets. **g** Network visualization of the predicted transcriptional regulatory networks enhanced by SU using TRRUST tool. **h** Violin plot showing the expression of *CDKN1A*, *PIM1*, *FOS* in CD4 T_EM_ between preSU and postSU groups in scRNA-seq. For the box plot within each violin plot, middle lines indicate median values, boxes range from the 25th to 75th percentiles. Significance in **a**, **b** and **h** was calculated using two-sided Wilcoxon test as implemented in the function “compare_means” with default parameters; **P < 0.01, ****P < 0.0001.

Lymphocytes, including CD4^+^ TCs and BCs, play important roles in the development of inflammation and autoimmunity^19^. Using CyTOF, we identified an increase in lymphocytes, especially TCs, after SU. Next, we explored functional marker expression across the corresponding cell lineages. Among the CD4^+^ TCs, the elevated markers included the Th17 markers CCR6, CXCR3, cell proliferating marker KI67, and apoptotic marker CD279. CD127 and GATA3 were downregulated in the postSU group (Fig. 3a, Supplementary Fig. 5a). In BCs, levels of CXCR3, CCR6, and the autoimmune-related BC (ABC) marker T-bet were increased after SU (Fig. 3b, Supplementary Fig. 5b). In addition, upregulation of CD38 was associated with an increase of PCs in BCs (Supplementary Figs. 4f and 5b). These results indicate that SU induces autoimmune-associated protein expression patterns.

We next explored the transcriptional patterns of SU and found that SU-induced immune activation of CD4^+^ TCs and BCs (Supplementary Fig. 5c, d). Accordingly, we performed an integrated comparative analysis of DEGs to determine subtype-specific gene signatures. The T-mito subset was the most affected by SU across TC subsets (Supplementary Fig. 5e). Notably, SU reduced *CD27* expression and increased the expression of genes related to inflammatory activation (*FOS*, *JUN*, *NFKBIA*), JAK signaling (*IFNG, IL2RB, TIMP1, GRB2, CCND3*), and DNA damage (*DDIT3*, *GADD45B*) in T-mito subset (Fig. 3c). The Treg and T_EM_ were the subsets most altered by SU in CD4^+^ TCs, while PCs were the most influenced by SU across BC subsets according to the upregulated DEG number (Supplementary Fig. 5e, f). By generating an UpSet plot of upregulated DEGs (Supplementary Fig. 5g), we found CD4 T_EM_ as a unique subset with increased levels of *PIM1* and *TNF*, which were involved in Th17 differentiation and autoimmune activation^20,21^. Notably, genes related to Th17 differentiation, including *PIM2*, *FOS*, *FOSB*, *JUN*, *NFKBIA*, were increased in Treg (Supplementary Fig. 5h). In addition, all BC subsets showed an increased expression of genes associated with an inflammatory state, including *NFKBIA*, *JUN*, and *COX5A*. Moreover, we identified subtype-specific expression patterns, including *KLF6* and *HSPA5* in ABCs; *XBP1*, *IRF4*, *JAK1*, and *IGHA1* in PCs; and *BACH2* and *IGHV4-34* in naive BCs (NBCs) (Fig. 3d).

To understand the biological implications of SU-related transcriptional changes, we performed GO and pathway enrichment analysis using the upregulated DEGs (Fig. 3e). The commonly upregulated DEGs after SU were enriched in protein processing and apoptosis. Notably, SU upregulated the activity of IFN-γ production, p38MAPK, and the IL-17 signaling pathway in CD4 T_EM_ and Treg (Fig. 3e). The JAK-STAT signaling pathway and adaptive immune system were overrepresented in T-mito cells from SU blood. Protein processing was also markedly enhanced in PCs (Fig. 3e). These processes and pathways are closely related to autoimmune activation^22^. SU also accelerated cellular senescence. In a supervised manner, we found that SU also enhanced the expression of immunomodulatory genes (*TIGIT*, *IL10RA*, *TGFB1*) in Treg (Supplementary Fig. 5i). In addition, we found that Th17 differentiation key transcription factor (TF) (*STAT3*) and *IL6R* were increased in Treg, while *IL6ST* was increased in CD4 T_EM_. The expression of *RORC* and *IL17RA* in CD4 T_EM_ was similar between the two groups (Supplementary Fig. 5j). To explore the possibility of SU-induced immune dysfunction underlying human diseases, we employed the COVID and DisGeNET databases to predict DEG-associated diseases. SU was found to increase COVID-19 risk, which was mainly attributed to the modulation of PCs and CD4 T_EM_ (Supplementary Fig. 5k). In addition, SU-enhanced DEGs were associated with an increased risk of autoinflammatory disease, Behcet syndrome, and lupus erythematosus.

To understand the transcriptional regulatory networks underlying SU, we used the TRRUST database^23^ to predict the core TFs regulating upregulated DEGs in lymphocytes (Fig. 3f). We found that TF activity was markedly upregulated after SU in CD4 T_EM_ and T-mito subsets. The main Th17-related TF *STAT3* was activated by SU, especially in CD4 T_EM_ (Fig. 3f)_._ In addition, two inflammation-related TFs, *NFKB1* and *RELA*, were upregulated by SU. Transcriptional regulatory network analysis indicated the unique upregulation of *CDKN1A*, *S100A9* and *TNF* in CD4 T_EM_ was regulated by *CEBPB* (Fig. 3g, h, Supplementary Fig. 5g). We tracked the networks and identified *STAT3* as the key TF regulating autoimmune-related genes (*PIM1*, *FOS* and *JUNB*) (Fig. 3g). In order to strengthen the conclusion, we next performed motif-enrichment to predict upstream regulators by using the RcisTarget tool, which is based on the methods previously implemented in i-cisTarget and iRegulon^24^. These results showed that *PIM1* was also regulated by the cis-regulatory module banded by *STAT3* and *STAT1* (Supplementary Fig. 6a). All the 10 TFs regulated *CDKN1A* expression. Notably, we found CD4 T_EM_ was the only subset to show an increase in *CDKN1A* and *PIM1* levels, and SU increased *FOS* expression in all subsets including CD4 T_EM_ (Fig. 3h, Supplementary Fig. 5g). Collectively, these findings indicate that SU reprograms the proteomic and transcriptional profile of circulating lymphocytes and induces an autoimmune-related phenotype.

### SU induces immune dysregulation in cytotoxic cells

**Fig. 4:**
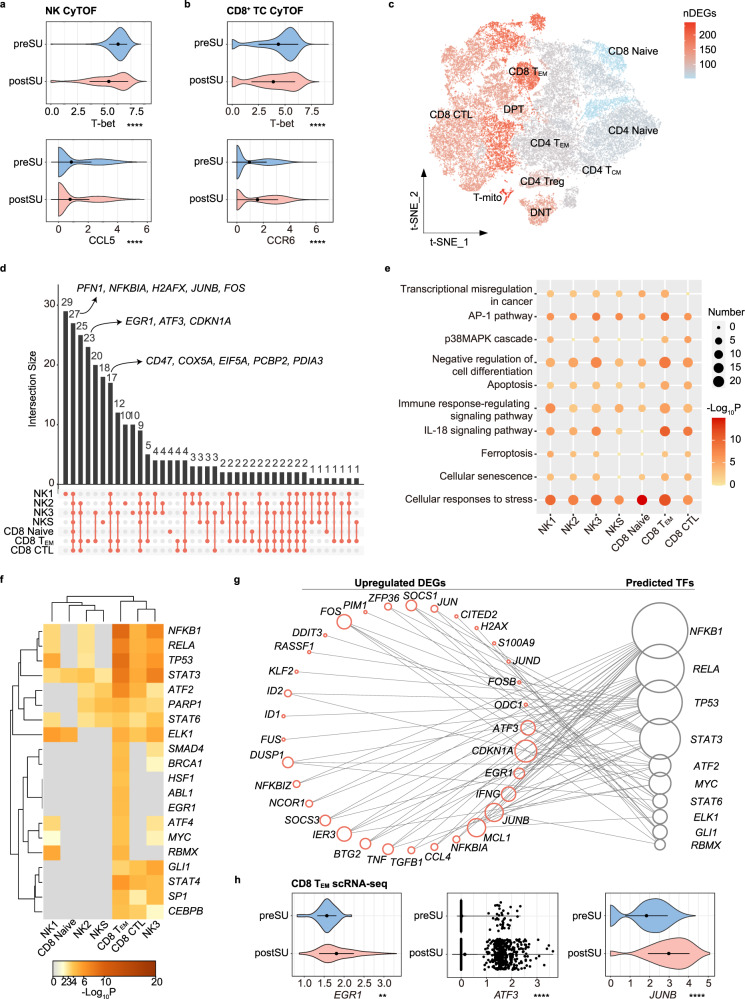
Changes in proteomic and transcriptional profiles of cytotoxic cells. **a** Violin plot showing the expression of T-bet and CCL5 in NK between preSU and postSU groups in CyTOF. **b** Violin plot showing the expression of T-bet and CCR6 in CD8^+^ TC between preSU and postSU groups in CyTOF. **c** Number of DEGs between preSU and postSU groups within each TC cluster projected onto the t-SNE map. **d** UpSet Plot showing the integrated comparative analysis of upregulated DEGs in cytotoxic cell subsets. **e** Representative GO biological process and pathways enriched in upregulated DEGs based on functional enrichment analysis in cytotoxic cell subsets. **f** The heatmap showing the enhanced activity of TFs predicted by TRRUST analysis in cytotoxic cell subsets. **g** Network visualization of the predicted transcriptional regulatory networks enhanced by SU using TRRUST tool. **h** Violin plot showing the expression of *EGR1*, *ATF3*, *JUNB* in CD8 T_EM_ between preSU and postSU groups in scRNA-seq. For the box plot within each violin plot, middle lines indicate median values, boxes range from the 25th to 75th percentiles. Significance in **a**, **b** and **h** was calculated using two-sided Wilcoxon test as implemented in the function “compare_means” with default parameters; **P < 0.01, ****P < 0.0001.

As the key goalkeeper in the antitumor and antivirus response, cytotoxic cells (including NK and CD8^+^ TCs) are influenced by a lack of sleep^25,26^. Using CyTOF, we found that SU affects NK differentiation. The CD57^+^ NK3 subset, with high cytotoxic and mature characteristics^27^, was shrank in postSU NK (Fig. 1i, Supplementary Fig. 4d). We next explored functional marker expression across the NK and CD8^+^ TC subsets. SU decreased the expression of T-bet (Fig. 4a, b), which is the key TF governing the differentiation and function of cytotoxic cells, and mildly reduced the expression of the NK-related chemokine CCL5 (Fig. 4a). Corresponding with the increase in CD8 T_EM_ frequency, effector marker CCR6 expression was also increased in CD8^+^ TCs (Fig. 4b).

We further explored the transcriptional signatures altered by SU. As shown in the volcano plot (Supplementary Fig. 6b, c), SU decreased *GZMB* expression and upregulated genes related to inflammatory activation (*FOS*, *JUN*, *NFKBIA*, *DUSP2*, *JAK1*, *PIM1*), tumor immunity (*CD47*, *PCBP2*, *EIF5A*, *PDIA3*, *EGR1*), and DNA damage (*H2AFX*, *DDIT3*, *GADD45B*). Additionally, SU upregulated *PFN1*, which is a negative regulator of the killing and migratory functions of cytotoxic cells^28^. We next assessed the cell subtype-specific gene signatures altered by SU. Based on the number of DEGs, TC and NK subsets showed heterogeneous transcriptional changes after SU, with the most affected being NK3 and CD8 T_EM_ cells (Fig. 4c, Supplementary Fig. 6d). We next generated an UpSet plot of upregulated DEGs and found that SU upregulated a set of genes including *PFN1*, *NFKBIA*, *H2AFX*, *JUNB*, and *FOS* (Fig. 4d). Moreover, in some subsets including CD8 T_EM_, SU increased the level of tumor immunity-related genes, including *CD47*, *PCBP2*, *EIF5A*, and *PDIA3* (Fig. 4d). These results indicate that cytotoxic cells in postSU blood show specific transcriptional profiles associated with the increase in inflammation and decrease in cytotoxic activity.

GO and pathway analysis of each subset demonstrated that the common SU-induced upregulated biological processes and pathways included cellular responses to stress, apoptosis, and AP-1 pathway (Fig. 4e). SU also mediated cell differentiation and cellular senescence. We found that these pathways were especially enhanced in CD8 T_EM_ subset. Through DEG-disease relationship analysis, we found that SU increased the risk of infection, such as influenza A or COVID-19 (Supplementary Fig. 6e). In addition, DEGs upregulated by SU were characterized by an increased risk of T-cell lymphoma, tumor immunity, and inflammatory disorder (Supplementary Fig. 6e).

We then employed TRRUST and RcisTarget to predict the core TFs involved in upregulated DEGs among cytotoxic cells (Fig. 4f). The CD8 T_EM_ and NK3 subset showed the highest upregulation of TF activity after SU. Four central TFs (*NFKB1*, *RELA*, *STAT3*, and *ATF2*)^29,30^ in the activation of inflammation were upregulated by SU. By tracking the transcriptional regulatory networks (Fig. 4d, g, h, Supplementary Fig. 6f), we found that the unique upregulation of *EGR1*, *ATF3*, *CDKN1A* in CD8 T_EM_ was regulated by *NFKB1*, while *STAT3, NFKB1* and *MYC* were identified as regulators of *JUNB*. In addition, SU upregulated *JUNB* in all subsets including CD8 T_EM_ (Fig. 4d, h). These results indicate that after SU, cytotoxic cells lose their immune activity and upregulate a phenotype associated with infection, tumor development, and inflammatory disorders.

### Myeloid cell plasticity reflects inflammation activation in SU blood

**Fig. 5:**
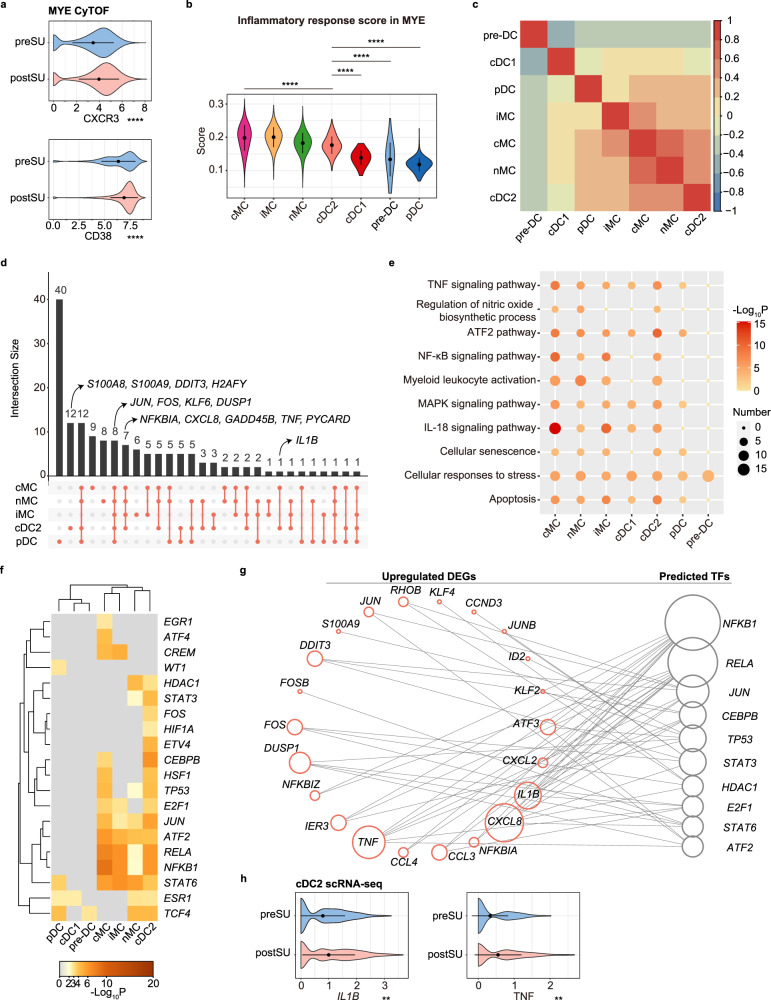
Changes in proteomic and transcriptional profiles of myeloid cells (MYEs). **a** Violin plot showing the expression of CXCR3 and CD38 in MYE between preSU and postSU groups in CyTOF. **b** Violin plot of inflammatory response scores for each MYE cluster, different clusters were represented in different colors. **c** Heatmap showing the correlation analysis of upregulated DEG in MYE subsets. **d** UpSet Plot showing the integrated comparative analysis of upregulated DEGs in MYE subsets. **e** Representative GO biological process and pathways enriched in upregulated DEGs based on functional enrichment analysis in MYE subsets. **f** The heatmap showing the enhanced activity of TFs predicted by TRRUST analysis in MYE subsets. **g** Network visualization of the predicted transcriptional regulatory networks enhanced by SU using TRRUST tool. **h** Violin plot showing the expression of *IL1B*, *TNF* in cDC2 between preSU and postSU groups in scRNA-seq. For the box plot within each violin plot, middle lines indicate median values, boxes range from the 25th to 75th percentiles. Significance in **a**, **b** and **h** was calculated using two-sided Wilcoxon test as implemented in the function “compare_means” with default parameters; **P < 0.01, ****P < 0.0001.

Human peripheral blood MYEs, including MCs and DCs, promote antigen presentation and inflammatory process. The increase in cMCs and decrease in nMCs frequency (Fig. 1j) indicated that monocyte differentiation is restrained by SU. In addition, the increase in cMCs percentage was considered to result from an enhanced innate immune response, as evident by the increase in CXCR3 and CD38 levels (Fig. 5a). Thus, we analyzed the transcriptome gene expression in MYEs after SU. In MCs, SU downregulated *EGR1* and *EGR2* (Supplementary Fig. 7a), which are negative regulators in the differentiation and inflammatory activation of myeloid cells^31,32^. Moreover, SU was associated with the upregulation of several inflammatory genes, including *TNF*, *IL1B*, *PYCARD*, *CXCL8*, *S100A8*, and *S100A9* (Supplementary Fig. 7a, b).

We next assessed the SU-altered transcriptional signatures of MYE subsets. As the inflammatory response score increased in the blood after SU (Fig. 2g), we measured the score of each MYE subpopulation. cMCs, which account for the majority of MYEs in peripheral blood, were the most inflammatory MYE, while cDC2 showed the highest inflammatory response score among DCs (Fig. 5b). To identify dissimilarities in MYE subgroup response to SU, we performed correlation analysis of the upregulated DEGs among each subset and found that cDC2 showed the strongest correlation with MC subsets in postSU blood (Fig. 5c). Via UpSet plots (Fig. 5d), we identified a set of genes whose expression (especially that of cDC2) was increased in MYE subsets, indicative of DNA damage (*DDIT3*, *H2AFY*, *GADD45B*) and inflammatory activation (*S100A8*, *IL1B*, *PYCARD*, *TNF*, *CXCL8*). Consistent with these findings, the CD11C^+^ myeloid cells expressing TNF and IL-1β were accumulated in postSU blood confirmed by the CyTOF results (Supplementary Fig. 7c, d).

Enrichment analysis further identified cellular responses to stress as the commonly upregulated process across cell subsets (Fig. 5e). Moreover, cDC2 and MCs shared several upregulated inflammatory pathways, such as the TNF, IL-18, NF-κB, ATF2, and MAPK signaling pathways. Similar to that in lymphocytes, SU also induced the hallmarks of cellular senescence in MYEs, suggesting a close relationship between SU and aging (Fig. 5e). We also found that SU induced the expression of SARS-CoV-2 associated molecule CD26 (encoded by *DPP4*) in the cDC1 subset (Supplementary Fig. 7e). Indeed, DEG-disease relationship analysis demonstrated that SU-induced upregulated DEGs were associated with an increased risk of infection (Supplementary Fig. 7f). SU also induced an increased predisposition to vascular inflammation, atherosclerosis, and autoinflammatory disease (Supplementary Fig. 7f). Functional analysis of SU-related DEGs among the MYE subsets further supports the role of SU in promoting proinflammatory pathways, apoptosis, and cellular senescence, all of which are processes contributing to inflammatory disorders.

The core TFs regulating upregulated DEGs in MYEs were then assessed (Fig. 5f). TF activity was the most upregulated after SU in the cDC2 and cMC subsets, with the NF-κB, ATF/CREB, and AP-1 families predominant in the top 20 TFs. SU upregulated several TFs that are associated with inflammation activation, including *NFKB1*, *RELA*, *JUN*, and *ATF2*. Moreover, the NF-κB family (*NFKB1*, *RELA*) was involved in the regulation of several inflammatory genes, including chemokines (*CCL3*, *CCL4*, *CXCL2*, *CXCL8*) and inflammatory cytokines (*IL1B* and *TNF*) (Fig. 5g). These results were confirmed by performing the motif-enrichment analysis with RcisTarget tool (Supplementary Fig. 7g). Notably, *IL1B* and *TNF* expression were especially elevated in postSU cDC2 (Fig. 5h). Altogether, these results demonstrate the key role of cDC2 in the SU-induced activation of inflammation.

### Aberrant cell-cell communication patterns support the immune dysfunction observed after SU

**Fig. 6:**
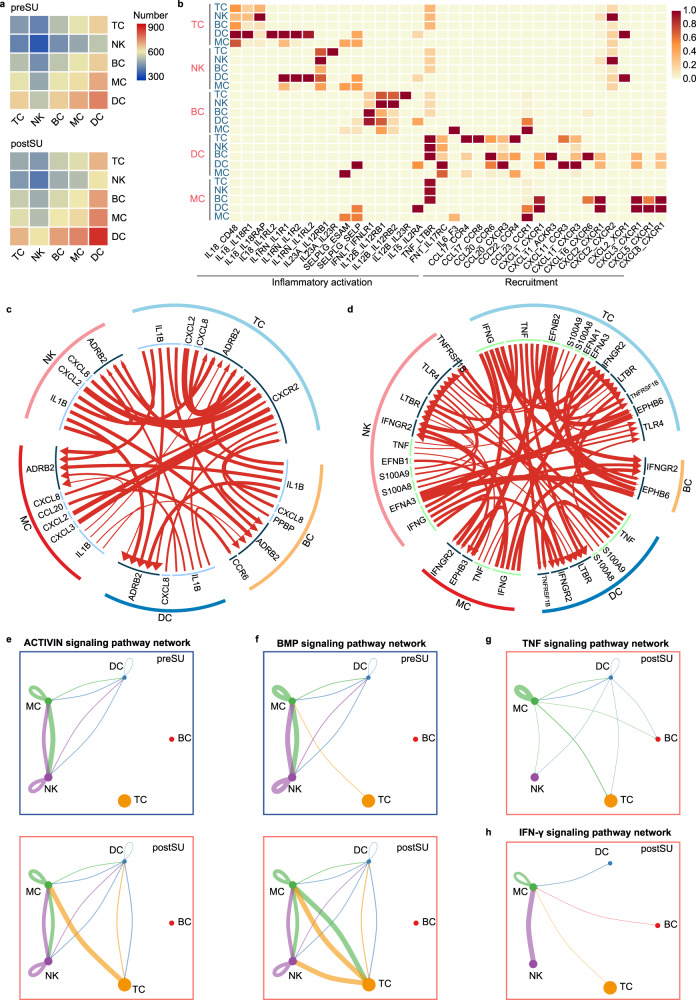
Enhanced cell chemotaxis and inflammatory activation in cell-cell communication in SU blood. **a** Heatmap depicting the number of possible interactions between the clusters analyzed in preSU and postSU groups. **b** Heatmap depicting selected cell-cell interactions enriched in postSU group but absent in preSU group. **c** Circle plot showing upregulation in cellular interaction of cytokine type predicted in iTALK package. **d** Circle plot showing upregulation in cellular interaction of other type predicted in iTALK package. **e** Circle plot showing the inferred ACTIVIN signaling networks. **f** Circle plot showing the inferred BMP signaling networks. **g** Circle plot showing the inferred TNF signaling networks in postSU group. **h** Circle plot showing the inferred IFN-γ signaling networks in postSU group.

Although immune dysfunction has been emphasized in studies related to poor sleep experiences, the specific SU-induced cell-cell interactions in the circulating immune system have not been investigated. Complex cellular responses start with the binding of a ligand to its cognate receptor and the activation of specific cell signaling pathways. To identify the cellular interactions affected by SU, we first explored cell-cell communication under the condition of SU using iTALK and CellChat^33,34^. We compared the number of interactions between cells among groups and found that the number of predicted interactions was increased in the postSU group compared with the preSU group (Fig. 6a).

Next, we identified potential cell-cell interactions in the blood and their alterations after SU; some were specific to this abnormal state (Fig. 6b). SU induced several interactions that were mainly involved in the inflammatory activation of lymphocytes to other cells and chemotaxis of MYEs to other cells. Specifically, the postSU group showed unique intercellular communication, including *IL18-IL18R1* between TCs and DCs; *IL23A-IL23R* between NKs and TCs; *IL12B-IL23R* between BCs and TCs; and *TNF-LTBR* in the crosstalk between MYEs and other cell types (Fig. 6b). Using iTALK, we found that SU increased the score of several combinations, which were shared by the preSU and postSU groups (Fig. 6c, d). The secreted cytokines—encoded by *IL1B*, *CXCL8*, *TNF*, and *IFNG*, all of which play a role in autoimmune and inflammatory diseases—may activate other immune cells expressing their cognate receptors (Fig. 6c, d). Moreover, the ephrin family (*EFNA1*, *EFNA3*, *EFNB1*, *EFNB2*) and their receptor EPH family (*EPHB3* and *EPHB6*) were over-represented in the intercellular interactions of the postSU group (Fig. 6d). As receptor tyrosine kinases, EPHs are involved in inflammation development and disease pathogenesis, including optic nerve injury and atherosclerosis^35^.

Using CellChat to explore the SU-induced signaling pathways, several pathways were found with abundant signaling interactions among cells, with a higher contribution by TCs in the postSU group compared to in the preSU group. These signaling pathways included the ACTIVIN, BMP, SELPLG, FASLG, and PARs pathways (Fig. 6e, f, Supplementary Fig. 8a–c), which play important roles in inflammatory processes and disorders. Moreover, CellChat detected TNF, IFN-γ, and THY1 signaling pathways in the postSU group (Fig. 6g, h, Supplementary Fig. 8d), but not in the preSU group, indicating that they were activated by SU. The EGF signaling pathway also showed heterogeneous output between the preSU and postSU groups, with MC in the preSU group and DCs in the postSU group (Supplementary Fig. 8e). Altogether, these findings reveal the specific interactions induced by SU and highlight the signaling pathways implicated in autoimmune and inflammatory disorders.

## Discussion

Here, we focused on the comparison of differences before and after SU all night in view of the subjects pairing. We evaluated the systemic effect of poor sleep on immunity in terms of cell type composition, subset-specific gene expression, enriched pathways, transcriptional regulatory networks, and cell-cell communication in an unbiased and global fashion. Using CyTOF and scRNA-seq, we obtained a comprehensive depiction of the impact of SU on various immune parameters at the single-cell proteomic and transcriptomic levels. The major findings of the present study include: 1) the expression of autoimmune-related markers and enriched pathways increased in effector CD4^+^ TCs, which induced a strong autoimmune predisposition following SU; 2) SU induced a pro-inflammatory status of peripheral blood by remodeling the composition of immune subsets, gene expression signatures, and cell-cell communication; 3) Cytotoxic cells lost their immune activity and exhibited a phenotype associated with infection, tumor development, and inflammatory disorders after SU; and 4) DNA damage-associated genes and aging-related processes were elevated following SU, indicating accelerated cellular senescence.

The dysregulation of immunity caused by poor sleep (SU and SL) involves the breakdown of immunologic self-tolerance^36,37^, thus exposing people with sleep disorders or night shift work to a higher risk of autoimmune diseases, such as systemic lupus erythematosus (SLE) and rheumatoid arthritis (RA)^4,38^. In an experimental model of SLE, sleep deprivation promoted the onset and progress of lupus in mice^37^. However, the detailed mechanisms underlying this causality have not yet been elucidated. Pathologic effector CD4^+^ TCs, including Th1 and Th17, and their cytokine production (IFN-γ, IL-17, TNF-α) have been implicated in inflammation and autoimmunity^19,39^. In this study, we obtained a comprehensive classification of immune subsets, with alterations in immune components at a high resolution and precision. Following SU, autoimmune-related genes (*JAK1*, *PIM1*, *TNF, IL6R*) and pathways (JAK-STAT, IL-17) were widely upregulated in lymphocytes, especially in the CD4 T_EM_ and T-mito subsets. In addition, SU upregulated autoimmune-related biomarkers (CXCR3, CCR6, *IGHV4-34*). CXCR3 contributes to the pathogenesis of RA by regulating TC recruitment and Th17/Treg balance^40^. In addition, CXCR3^+^ BCs, recruited by proinflammatory IL-17^+^ cells, induce macrophage polarization in human hepatocellular carcinoma^41^. Apart from promoting chemotaxis, the CCR6/CXCL20 axis induced Th17/Treg imbalance and orchestrates multiple autoimmune diseases^42^. As for BCs, early CCR6 expression modulates germinal center kinetics and is crucial for efficient antibody responses^43^. Furthermore, CCR6 expression on BCs is upregulated in SLE patients^44^. IGHV4-34 is associated with reactivity against self-epitopes in autoreactive BCs. Such autoantibody-producing IGHV4-34^+^ BCs are enriched in the blood of SLE patients and the synovium of RA patients^45,46^. These highly expressed genes and markers that have been confirmed to be involved in the onset of autoimmune diseases probably account for the autoimmune susceptibility of sleep-deprived populations.

Aging is accompanied by subtle but broad changes in the immune system that increase susceptibility to infections, cancer, and other age-related diseases^47,48^. Recently, we elucidated the aging immune landscape by multimodal studies, and found that it is characterized by TC polarization towards memory and cytotoxic phenotypes, along with increased expression of inflammatory genes and SARS-CoV-2-related genes (including *DPP4*)^17^. A link between poor sleep experiences and accelerated cellular aging was initially demonstrated in animal experiments. In sleep-deprived rats, cellular stress and oxidative DNA damage were observed in multiple organs and plasma^49^. Moreover, the JAK/STAT pathway regulates cellular senescence and some aging-associated alterations; indeed, the JAK1/2 inhibitor ruxolitinib alleviates age-related bone loss and adipose tissue inflammation in mice and rescues some premature aging phenotypes in progeria mouse models^50,51^. However, animal models fail to summarize the human immune environment adequately. Due to technical limitations previously, high-dimensional cellular and molecular mechanisms underlying the upregulated senescence induced by poor sleep are lacking. Here, we extended these findings to human blood to the best of our knowledge, as single-cell transcriptomics revealed SU upregulated DNA damage-related gene expression (*H2AX, GADD45B, DDIT3*), JAK-STAT pathway activation, SASP, autophagy, and apoptosis in lymphocytes and MYEs. Recently, the important role of CD38 in aging has also been emphasized. Senescence-induced inflammation promotes the accumulation of CD38 in immune cells, decreasing NAD^+^ levels^52^. Similarly, we found that SU increased CD38 marker expression in MYEs, which may reduce NAD^+^ levels and promote cellular senescence. In addition, in mice ovary, cell apoptosis and DNA damage were reported to be enhanced by light-exposure at night^53^. Overall, the loss of sleep durations and light exposure during SU promotes DNA damage and cellular senescence in the transcriptome, presumably accelerating aging.

SU all night to work and SL have been reported to be associated with increased cancer and infection susceptibility^54,55^. Shorter sleep durations or poor sleep quality may be the facets associated with the development of aggressive tumor characteristics^56^. In addition, it’s reported that light exposure at night could reduce bactericidal activity of blood^57^ and accelerate tumor growth in animals^58^. Experiments on sleep-restricted mice demonstrated that the oncogenicity of SL is partly attributable to decreased cytotoxic cells (NK and CD8^+^ TCs) in the tumor environment and blood^25^. However, what we know about immune cells primarily depends on previously described markers for pooled cell populations. Herein, by using single-cell technologies, we described the key cellular and molecular alterations involved in impaired cytotoxic function and increased inflammation. Within the NK populations, CD57^+^ NKs are distinguished by their mature phenotype, high cytotoxic capacity, and cancer surveillance function^27^. Consistently, high frequencies of peripheral or tumor-associated CD57^+^ NKs have been linked to reduced disease severity and better outcomes in cancer patients^59,60^. The CD57^+^ NK population shrank after SU, suggesting blocked differentiation and maturation of NKs. Moreover, the expressions of key regulatory factors, such as *T-bet* and *PFN1* that regulate cell-killing and migration of cytotoxic cells, were altered. PFN1 is a negative regulator of the cytotoxic cell-mediated elimination of target cells, and in vitro downregulated PFN1 promotes cytotoxic TC invasion into mimical tumor environment^28^. In addition, increased PCs frequency and cell communication may act as compensatory mechanisms for protection from infection, but these changes also promote autoimmunity and inflammation on the other hand. Interestingly, the proinflammatory TF families AP-1 and NF-κB, along with their target genes, are reported to be highly expressed across tumor samples, and promote the development of oncogenic processes such as angiogenesis, cell migration and pro-tumoral inflammation^61^. The elevated AP-1 and NF-κB network after SU might expose people to higher risk of cancers. Therefore, the reduced cytotoxic cell populations along with the altered gene expression jointly form an impaired immune defense against infections and tumors in blood environment after SU.

In the absence of an infectious challenge, adequate sleep contributes to inflammatory homeostasis; however, poor sleep induces an inflammatory milieu, as evidenced by the upregulation of pro-inflammatory cytokine production and NF-κB activation^62,63^. It is generally accepted that serum levels and intracellular production of TNF-α and IL-1β are elevated after SL^62,64^. Single-cell technologies open new ways in many research fields. More importantly, they are particularly important for analyzing the impact of poor sleep on human cells. In addition to the common cytokines above, we provided broader proteomic and transcriptomic signatures in blood environment after SU. In this study, we found that inflammatory cMC and cDC accounted for higher proportions in CD45^+^ cells, and TC cellular functions skewed towards effector phenotypes following SU. The upregulated inflammatory cytokines (*IL1B*, *TNF*), chemokines (*CCL3*, *CCL4*, *CXCL8*), TFs (NF-κB, ATF/CREB, and AP-1 family), and pathways (NF-κB, AP-1, and MAPK) involved in inflammation, demonstrated the inflammatory status induced by SU. In mice brain, the elevated DNA-binding activity of NF-κB after sleep deprivation was reported to be mediated by adenosine-induced NF-κB nuclear translocation^65,66^. Moreover, the NF-κB- and AP-1-dependent networks mediated microglia activation and inflammatory tissue destruction^67^. *EGR1*, a repressive regulator of the pro-inflammatory activities of myeloid cells by binding to inflammatory enhancers^31^, was found downregulated after SU. Moreover, we identified the key role of cDC2 in SU-induced inflammation in terms of inflammatory response score, gene expression profiles, GO enrichment and regulatory networks. It is known that sleep deficiency is associated with an increased risk of various inflammatory diseases, such as diabetes, atherosclerosis, and neurodegeneration^9–11^. In addition, mice showed the exacerbation of inflammatory responses and neuroinflammatory damage after dim light exposure at night^68^. Thus, our finding of the pro-inflammatory effect of SU offers a potential explanation for its immune mechanisms.

By simulating the condition of SU all night, we focused on the impact of poor sleep on the immune system. There are several important limitations in the study design. First, we didn’t perform a 2-week interval between two experimental nights in this study. Considering the objective of this study, which is exploring the impact of staying up and acute sleep loss on immune system, the 2-week interval may present confounding factors, including changes in diet, mood state, and menstrual cycle. We believe that our efforts can minimize the influence of confounding factors such as food consumption and ambulation during the study. Second, the design of this study didn’t employ with a randomized and balanced order of conditions. Third, considering the particularity and high cost of single-cell technologies, the sample size included in this study is small.

In summary, we represent the first high-dimensional single-cell analysis to obtain a comprehensive human circulating immune cells atlas of poor sleep by employing CyTOF and scRNA-seq. In describing key cellular and molecular differences before and after SU, such as effector CD4^+^ TC, NK3 and cDC2 subsets, we elucidate the potential contributions of SU—enhancing the inflammatory, cellular senescence, and autoreactive signatures—to immune dysfunction. The first application of single-cell technologies we conducted in this study provides a comprehensive profile of the effect of poor sleep on the immune system and expands our knowledge of related pathologic conditions.

## Materials and methods

### Human subjects and ethics statement

Six healthy participants (aged 39–52, BMI 19–25, 3 males and 3 females) were recruited for the study (Supplementary Table 1). To be eligible for study participation, subjects met the following inclusionary criteria: age range from 35 to 55 years; physical and psychological health; no clinically significant abnormalities in blood chemistry; regular sleep habits and a steady sleep time of ~8 h (22.00-06.00). Exclusion criteria included any physiological or psychiatric pathology, medication, smoking, obesity, binge drinking, or excessive caffeine use (>3 cups per day), extreme morningness, extreme eveningness, sleep or circadian disorders. The study was approved by the Ethics Committee of Zhongshan Ophthalmic Center, China. Written informed consent was obtained from all participants and all procedures were performed according to the Declaration of Helsinki.

### Study protocol

When screening the volunteers (two weeks before the study), we asked them to spend a habituation night in the laboratory at Zhongshan Ophthalmic Center to make sure they met the requirements of the study and to acclimate them to the environment. Then, participants were required to follow a strict sleep-wake schedule (22:00-06:00) two weeks prior to the start of the experiment by checking the sleep logs. All participants completed the two conditions (normal sleep on day 1 and staying up (SU) all night on day 2, identified here as preSU and postSU). After 8 h of sleep (22:00-06:00) on one habituation day (day 1) in the laboratory, the 24 h periods of sleep loss were conducted began at 06:00-07:00 and lasted 24 hours (day 2). Throughout the 2-day study, laboratory conditions were highly controlled in terms of environmental conditions, including ambient light and temperature. Except for no light in the 8 h of normal sleep time (22:00-06:00) on day 1, the light was kept on to simulate the real condition of SU all night. In order to parallel normal behavior, subjects were outdoors at least three times in the daytime (06:00-18:00) under surveillance of a research assistant. Participants were restricted from exercising or engaging in strenuous activities, while they were allowed to do nonvigorous activities (reading, watching television, playing calm games, surfing the internet, talking with each other) and consume food and drink. Specially, participants were asked to keep quiet and weren’t allowed to consume food, watch television, walk around and talk with each other during 22:00-06:00. Under constant monitoring of three study staff members, participants weren’t allowed to close their eyes to ensure wakefulness on day 2. After the study, the participants were assessed for their mental state and found to be anxious and sleepy subjectively, which indicated that humans didn’t easily habituate to SU.

After participants sitting for half an hour, blood samples were obtained at the beginning of the experiment (06:00-07:00) and after 24 h of sleep loss. PBMCs were isolated by standard density gradient centrifugation. Trypan Blue was used to identify the viability and quantity of PBMCs in single-cell suspensions; cell viability of all samples exceeded 90% with >1 × 10^7^ viable cells. A proportion of PBMCs was allocated for scRNA-seq analysis and another was used for mass cytometry. To elucidate how SU affects cellular frequency, we measured single-cell protein expression using a 38-marker CyTOF panel (*n* = 12; Supplementary Table 2A). In scRNA-seq, a total of 12 libraries were sequenced, and 96,465 cells (preSU: 45,797 cells; postSU: 50,668 cells) were collected for subsequent analyses. By combining CyTOF and scRNA-seq, we created a comparative framework detailing the impact of SU on cell type distribution, gene expression changes, and cell-cell interaction analyses.

### scRNA-seq

#### scRNA-seq data alignment, processing, and sample aggregation

Single-cell suspensions were converted to barcoded scRNA-seq libraries using the Chromium Single Cell 5′ Library (10X Genomics, Genomics chromium platform Illumina NovaSeq 6000), Gel Bead and Multiplex Kit, and Chip Kit (10X Genomics). The Chromium Single Cell 5′ v2 Reagent Kit (120237; 10X Genomics) was used to prepare single-cell RNA libraries according to the manufacturer’s instructions. FastQC software was used to check library quality. Preliminary processing of sequenced data was performed using CellRanger software (version 4.0; 10X Genomics). The count pipeline in the CellRanger Software Suite was applied to demultiplex and barcode the sequences. Based on the calculation of the single-cell expression matrix by CellRanger, filtration, normalization, dimensionality reduction, clustering, and differential gene expression analysis were conducted using the Seurat package (version 3.0)^69^. Before filtration using the Seurat package, we removed the cell population expressing *HBB*, *HBA1*, and several light and heavy chain transcripts, which are considered red blood cell-contaminated^17^. Next, cells with fewer than 200 genes detected and a mitochondrial gene ratio of greater than 15% were excluded. A total of 96,465 cells (preSU, 45,797 cells; postSU, 50,668 cells) were analyzed after quality control.

#### Dimensionality reduction and clustering analysis

The “NormalizeData” function was used to log-normalize the counts of each cell (1+ counts per 10,000). The top 10 most variable genes were extracted by the “FindVariableGenes” function in Seurat with the default parameters. Dimensionality was achieved by “RunPCA” function. The “FindNeighbors” and “FindClusters” functions were used to identify significant clusters at an appropriate resolution. Cells were visualized using a 2-dimensional t-SNE algorithm based on the “RunTSNE” function. The function “FindAllMarkers” was used to identify marker genes of each significant cluster.

#### Differential expression analysis

For each cell type between different groups, differential expression analysis was performed using the Wilcoxon rank-sum test as implemented in the “FindMarkers” function of the Seurat package (version 3.0). Before performing differential expression analysis, the cell types that were missing or had fewer than three cells in the two groups were filtered out. A SU-related DEG dataset was established (*P* value <0.05, |Log_2_FC| >0.25) after identification of DEGs between groups. In some cases, we compared the expression of specific genes (like immunomodulatory genes) in the two groups with lower criteria (*P* value <0.05, |Log_2_FC| <0.25). The detailed DEGs dataset was provided in Supplementary Data 1–6.

#### Gene functional annotation

The Metascape webtool (www.metascape.org) was used to conduct GO biological process and pathway analysis as well as the DisGeNET and COVID databases, allowing us to visualize the functional patterns of DEGs and conduct statistical analysis^70^. We visualized the top 10 of 30 GO biological process and pathway terms associated with SU enriched among participants and cell types, which were drawn using the ggplot2 R package.

#### Scoring of biological processes

Individual cells were scored for their expression of gene signatures representing certain biological functions by calculating the average normalized expression of corresponding genes. Functional signature with the full gene list is provided in Supplementary Data 7. For instance, we determined the inflammatory response score by calculating the average expression of genes in the GO term “inflammatory response” (GO: 0006954). The SASP score was measured by the upregulation of the Reactome Gene Sets “Senescence-Associated Secretory Phenotype (SASP)” (R-HSA-2559582). ROS-related genes were obtained from the CTD Gene-Chemical Interactions dataset “Reactive Oxygen Species” (D017382).

#### Transcription factor-target gene network analysis

Core regulatory transcription factors were predicted based on the scRNA-seq data. As a web-based portal, Metascape (www.metascape.org) was used to conduct TRRUST analysis with the input of upregulated DEGs^70^. In addition, TF-binding motifs were identified via the RcisTarget R package (version 1.8.0) of the SCENIC workflow using default parameters^24,71^. RcisTarget was used to identify enriched transcription factor-binding motifs and to predict candidate target genes based on the hg19 RcisTarget database containing motifs with genome-wide rankings. Only the transcription factor targets with high-confidence annotation were selected and the transcriptional regulatory networks were visualized with Cytoscape (version 3.8.2)^72^.

#### Determination of cell-cell interactions

Based on the scRNA-seq data, cell-cell communication between different cells was predicted with the help of iTALK (https://github.com/Coolgenome/iTALK) and CellChat (https://github.com/sqjin/CellChat) R packages^33,34^. Communication was considered absent if the ligand and receptor were not detected; thus, only receptors and ligands expressed in at least 10% of specific cells were further analyzed. TBtools (www.tbtools.com) was applied to normalize the data and draw a heatmap. The differences in cellular communication between different groups were also analyzed and visualized using iTALK. In addition, CellChat, an R package that quantitatively analyzes intercellular communication networks and predicts major signaling inputs and outputs for cells, was used to analyze and visualize signaling pathway networks.

### Mass cytometry

#### Antibodies and reagents

Monoclonal anti-human antibodies for mass cytometry (Supplementary Table 2A) were either acquired preconjugated to heavy metal isotopes (Fluidigm, South San Francisco, CA) or conjugated via the MaxPar X8 Chelating Polymer Kit (Fluidigm).

#### Live cell barcoding and surface staining

A live cell barcoding methodology was applied to decrease inter-sample staining variability, sample handling time, and antibody consumption. The barcoded and combined samples were stained with 0.5 μmol/L viability dyes (cisplatin-195pt; 201064; Fluidigm), vortexed for 2 min at room temperature (RT), and then the reaction was terminated using Maxpar Cell Staining Buffer on a rotating shaker (400 rcf) at RT. The cells were then washed and fixed in 1.6% paraformaldehyde in PBS for 10 min at RT on a rotary shaker (500 rpm). The cells were resuspended in pre-cooled Maxpar Cell Staining Buffer to slow the fixation reaction, followed by washing twice with PBS/bovine serum albumin and once with double-distilled water. Finally, the cells were resuspended in 400 μL surface antibody mixture and incubated at 37 °C for 30 min on a rotating shaker (500 rpm) for surface staining. The samples then stored in freshly diluted 2% formaldehyde in PBS containing 0.125 nmol/L iridium 191/193 intercalator (Fluidigm, 201192) at 4 °C overnight.

#### Intracellular factor staining

The cells were washed twice with permeabilization buffer [0.5% saponin, 2% bovine serum albumin, and 0.01% sodium azide (all Sigma-Aldrich) in PBS]. Cells were resuspended in 400 μL intracellular antibody mixture in permeabilization buffer for 1 h at 4 °C on a rotary shaker (500 rpm). The samples were then washed, the supernatant removed, and the cells resuspended in 1X iridium intercalator solution (Fluidigm) overnight. Finally, the sample was washed twice with PBS/bovine serum albumin and once with double-distilled water before acquisition.

#### Mass cytometry acquiring, processing and quality control

CyTOF data were obtained from a SuperSampler fluidics CyTOF2 system (Victorian Airships, Alamo, CA), at an event rate of <400 /s, and then normalized with Helios normalizer software (version 6.7.1016; Fluidigm). Quality control and tuning of the CyTOF2 mass cytometer (Fluidigm) was performed daily. Cytobank software (version 7.0; https://mtsinai.cytobank.org) was used to deconvolute barcoded samples and filter cross-sample doublets. Based on event length and live cell (195Pt) and DNA (191Ir and 193Ir) channels, Cytobank was used to sequentially remove dead cells, calibration beads, debris, and barcodes of CD45^+^ PBMCs. The FCS files were then exported for downstream analysis. All cytometry data were transformed with an inverse hyperbolic sine (arcsinh) function (mass cytometry: cofactor of 5) using R. We analyzed 240,000 cells with an average of 20,000 cells per sample.

#### Mass cytometry visualization and clustering

We used FlowCore to read and process the FCS files for further analysis. For samples with more than 20,000 cells, we randomly selected 20,000 cells to ensure that samples were equally represented. The CATALYST R package was used to integrate data for analysis. All FlowSOM-based clustering and subclustering were performed on the dataset to identify specific populations^73^. Mass cytometry datasets derived from all individuals for each cell type were created for analysis. We created downsampled datasets of 64,813 T cells, 24,113 NK cells, 6,807 B cells, and 24,267 MYEs in the preSU group, and 68,590 T cells, 24,771 NK cells, 7,174 B cells, and 19,465 MYEs in the postSU group for analysis. The detailed cell counts are provided in Supplementary Data 8.

#### Differential abundance (DA) analyses

We plotted the abundance changes and median expression of markers between preSU and postSU groups using boxplots with jittered points of the sample-level cluster proportions overlaid as well as heatmaps with z-score normalization. We used the diffcyt R package to perform differential analyses of the CyTOF data. Accounting for the subjects pairing, we set the mixed model formula using the *createFormula()* function. Then, we used the “diffcyt-DA-GLMM” method for DA analysis.

### Statistics and reproducibility

For analyzing cluster abundance, groups were compared using two-tailed paired *t*-tests, and GraphPad Prism (version 8.0.2; GraphPad Software Inc., La Jolla, CA) was used for data analysis and presentation. For comparing percentage changes in subset composition between groups in CyTOF, adjusted *P* values for each cluster was calculated using the “diffcyt-DA-GLMM” method as implemented in the “diffcyt” function of diffcyt R package in view of the subjects pairing. For comparing the level of markers or genes between groups, *P* value was calculated using two-sided Wilcoxon test as implemented in the function “compare_means” of ggpubr R package with default parameters. In calculating the GO biological process, pathway, COVID and DisGeNET terms, *P* values were derived by a hypergeometric test with the default parameters in Metascape webtool. Details of the size of biological replicates and the assays are given in each figure legends. *P < 0.05, **P < 0.01, ***P < 0.001, ****P < 0.0001.

### Reporting summary

Further information on research design is available in the Nature Research Reporting Summary linked to this article.

## Supplementary information

Supplementary Information

Description of Additional Supplementary Files

Supplementary Data 1

Supplementary Data 2

Supplementary Data 3

Supplementary Data 4

Supplementary Data 5

Supplementary Data 6

Supplementary Data 7

Supplementary Data 8

Supplementary Data 9

Reporting Summary

## Data Availability

The data that support the findings of this study are available from the corresponding author upon request. The scRNA-seq data is deposited in the Genome Sequence Archive in BIG Data Center, Beijing Institute of Genomics (BIG, https://bigd.big.ac.cn/gsa-human/), Chinese Academy of Sciences, under the Project Accession No. PRJCA004314 and GSA Accession No. HRA000604. Experimental protocols and the data analysis pipeline used in our work follow those described on the 10X Genomics and Seurat official websites. The analysis steps, functions, and parameters used are described in detail in the Materials and Methods section. The source data underlying plots shown in figures are provided in Supplementary Data 9.
