# Supplementary material for: Effects of poor sleep on the immune cell landscape as assessed by single-cell analysis
Source: Commun Biol. 2021 Nov 25;4:1325. doi: 10.1038/s42003-021-02859-8 (PMC8617259; doi:10.1038/s42003-021-02859-8)
Supplement: Supplementary file 1 — Supplementary Information [file 42003_2021_2859_MOESM1_ESM.pdf]

Supplementary Materials

Supplementary Figure

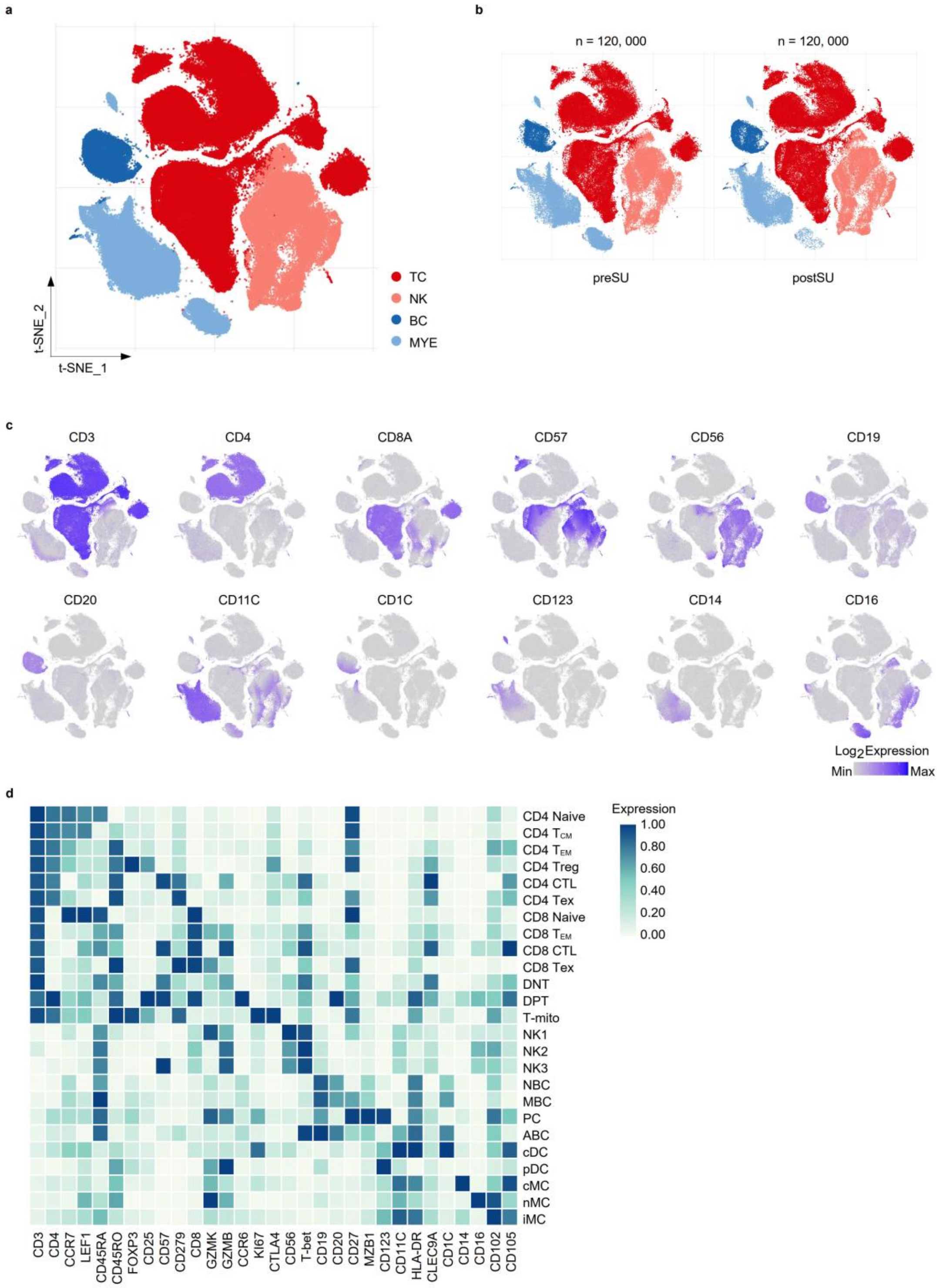

Supplementary Figure 1. The clustering strategy in CyTOF.

a t-SNE projection showing TC, NK, BC and MYE in CyTOF. **b** t-SNE projections segregated into preSU and postSU groups. **c** t-SNE projections showing the expressions of canonical markers. **d** Mean population expression levels of 31 type markers used for t-SNE visualization and FlowSOM clustering.

The full names of the 25 cell types in CyTOF are as follows: CD4 Naive: $CD4^+$ naive T cell; CD4 T_{CM}, central memory $CD4^+$ T cell; CD4 T_{EM}, effector memory $CD4^+$ T cell; CD4 Treg, regulatory $CD4^+$ T cell; CD4 CTL, cytotoxic $CD4^+$ T cell; CD4 Tex, exhausted $CD4^+$ T cell; CD8 Naive: $CD8^+$ naive T cell; CD8 T_{EM}, effector memory $CD8^+$ T cell; CD8 CTL, cytotoxic $CD8^+$ T cell; CD8 Tex, exhausted $CD8^+$ T cell; DNT, double-negative T cell; DPT, double-positive T cell; T-mito, mitotic T cell; NK1, $CD16^-$ $CD56^{bright}$ NK; NK2, $CD16^+$ $CD56^{dim}$ $CD57^-$ NK; NK3, the $CD16^+$ $CD56^{dim}$ $CD57^+$ late NK; NBC, naive B cell; MBC, memory B cell; PC, plasma cell; ABC, age-associated B cell; cMC, classical monocyte; nMC, nonclassical monocyte; iMC, intermediate monocyte; cDC, conventional DC; pDC, plasmacytoid DC.

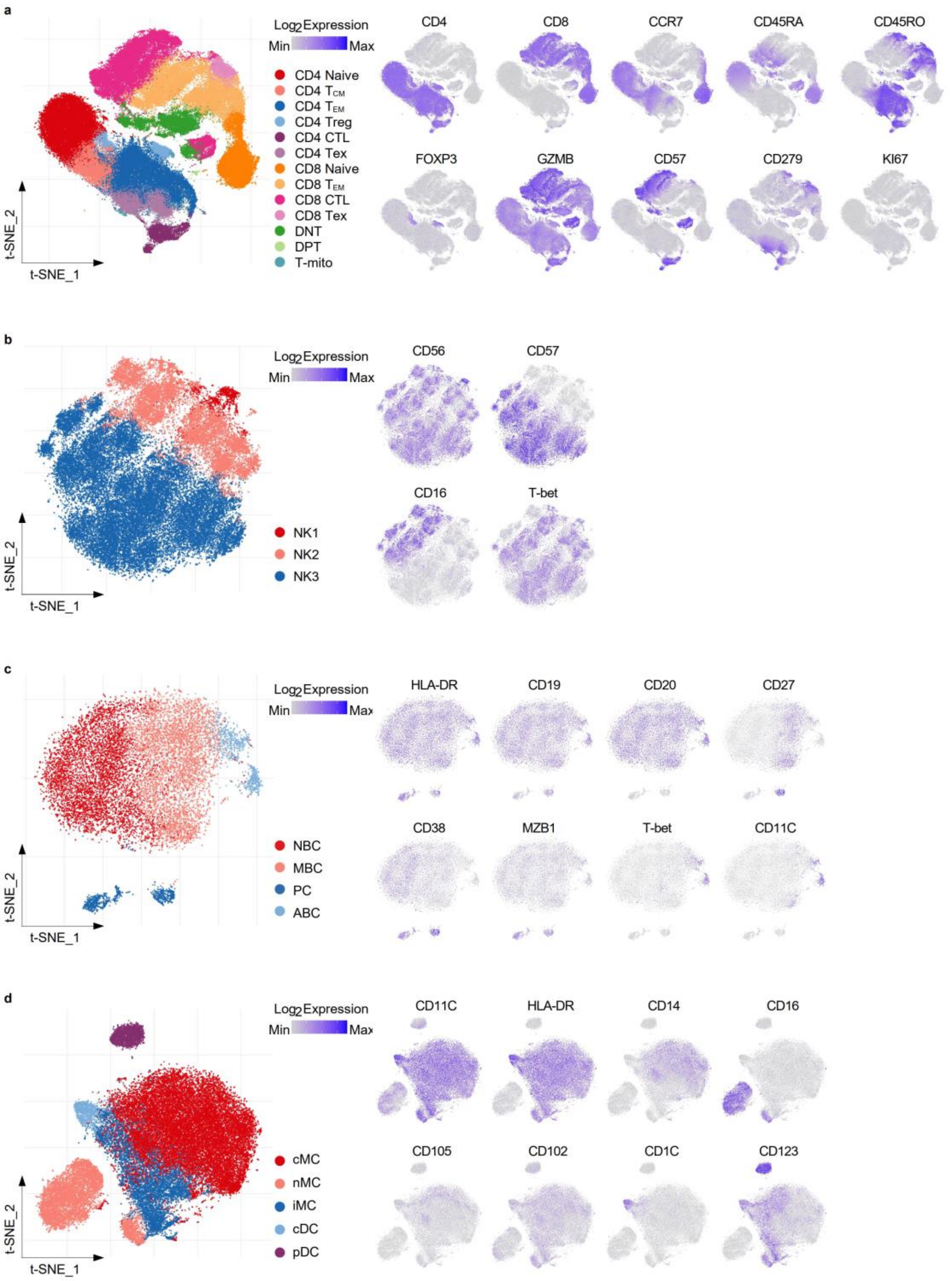

Supplementary Figure 2. Re-clustering of major immune cell populations.

(a) The clustering strategy of TC subsets. t-SNE projections showing TC subsets and canonical markers. **(b)** The clustering strategy of NK subsets. t-SNE projections showing NK subsets and canonical markers. **(c)** The clustering strategy of BC subsets. t-SNE projections showing BC subsets and canonical markers. **(d)** The clustering strategy of MYE subsets. t-SNE projections showing MYE subsets and canonical markers.

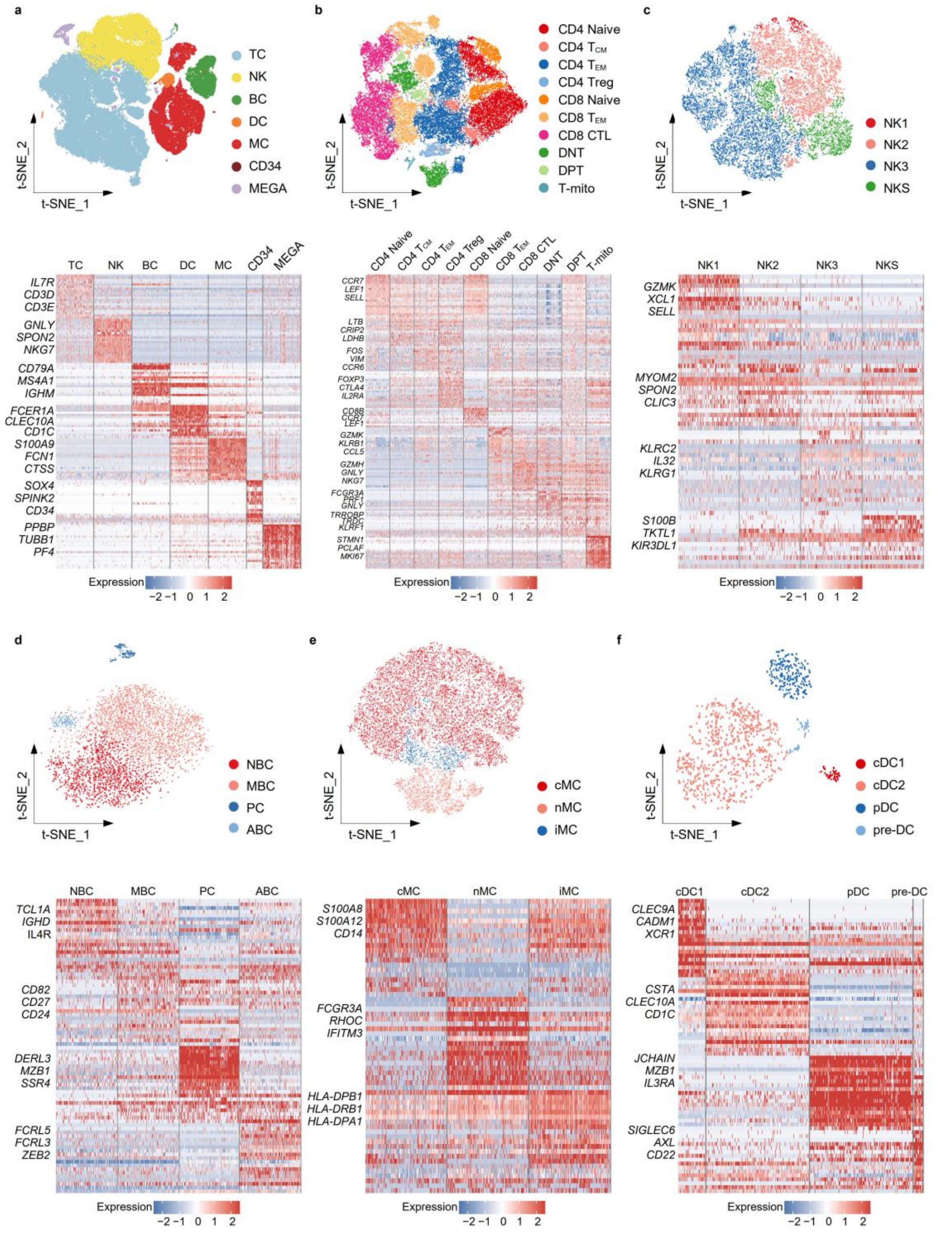

Supplementary Figure 3. Clustering strategy in scRNA-seq.

The clustering strategy of major immune cell populations (**a**) identifying seven cell types (top) based on the scaled expression heatmap of discriminative gene for each cluster (bottom). Then TC (**b**), NK (**c**), BC (**d**), MC (**e**), DC (**f**) was re-clustered and identified classical subsets (top) based on the scaled expression heatmap of discriminative gene for each cluster (bottom). Color scheme is based on z-score distribution from −2 (blue) to 2 (red).

The full names of the 25 cell types in scRNA-seq are as follows: CD4 Naive: $CD4^+$ naive T cell; CD4 T_{CM}, central memory $CD4^+$ T cell; CD4 T_{EM}, effector memory $CD4^+$ T cell; CD4 Treg, regulatory $CD4^+$ T cell; CD8 Naive: $CD8^+$ naive T cell; CD8 T_{EM}, effector memory $CD8^+$ T cell; CD8 CTL, cytotoxic $CD8^+$ T cell; DNT, double-negative T cell; DPT, double-positive T cell; T-mito, mitotic T cell; NK1, *FCGR3A*$^-$ *NCAM1*^{bright} NK; NK2, *FCGR3A*$^+$ *NCAM1*^{dim} *B3GAT1*$^-$ NK; NK3, the *FCGR3A*$^+$ *NCAM1*^{dim} *B3GAT1*$^+$ late NK; NKS, *S100B*$^+$ NK; NBC, naive B cell; MBC, memory B cell; PC, plasma cell; ABC, autoimmunity-associated B cell; cMC, classical monocyte; nMC, nonclassical monocyte; iMC, intermediate monocyte; cDC1, conventional DC type 1; cDC2, conventional DC type 2; pDC, plasmacytoid DC; pre-DC, precursor DC.

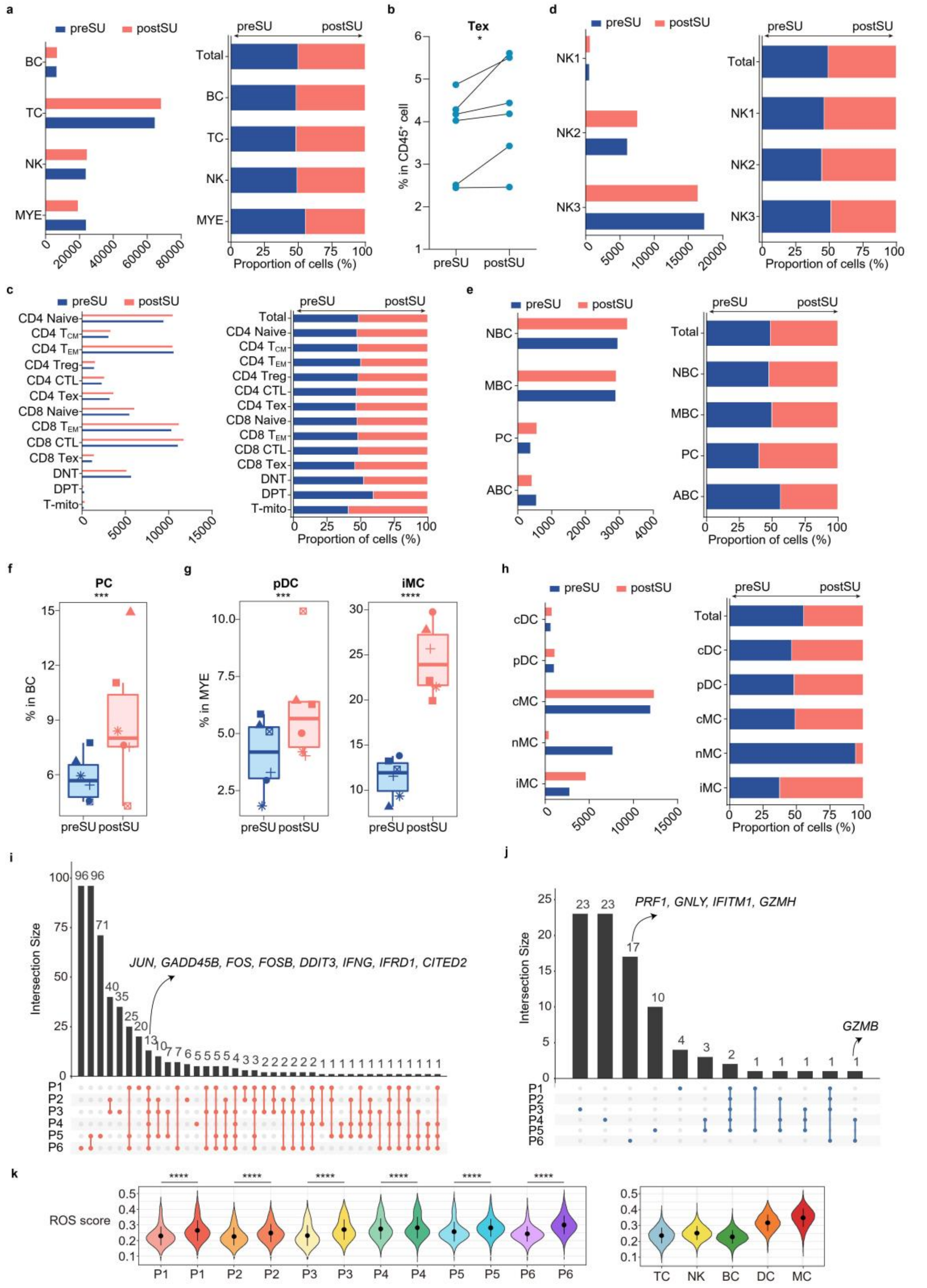

Supplementary Figure 4. Changes in cell proportions and transcriptional state among subjects after SU.

a Bar plots highlighting cell abundances across 4 main immune cell lineages for the preSU and postSU groups. **b** The percentage of Tex in immune cell between preSU and postSU groups (n = 6 /group). Bar plots highlighting cell abundances across 13 TC-subclusters **(c)**, 3 NK-subclusters **(d)**, 4 BC-subclusters **(e)**, 5 MYE-subclusters **(h)** for the preSU and postSU groups.

f The percentage of PC in BC between preSU and postSU groups (n = 6 /group). **g** The percentage of pDC and iMC in MYE between preSU and postSU groups (n = 6 /group). **i** UpSet Plot showing the integrated comparative analysis of upregulated DEGs among subjects. **j** UpSet Plot showing the integrated comparative analysis of downregulated DEGs among subjects. **k** Violin plot of ROS score for each sample and immune cell lineage. Different participants were represented in different colors, with darker colors representing after SU. For the box plot within each violin plot, middle lines indicate median values, boxes range from the 25th to 75th percentiles. Significance in b was calculated using two-tailed paired t test; significance in f and g was calculated using the "diffcyt-DA-GLMM" method as implemented in the “diffcyt” function in view of the subjects pairing; significance in k was calculated using two-sided Wilcoxon test as implemented in the function “compare_means” with default parameters; *P < 0.05, ***P < 0.001, ****P < 0.0001.

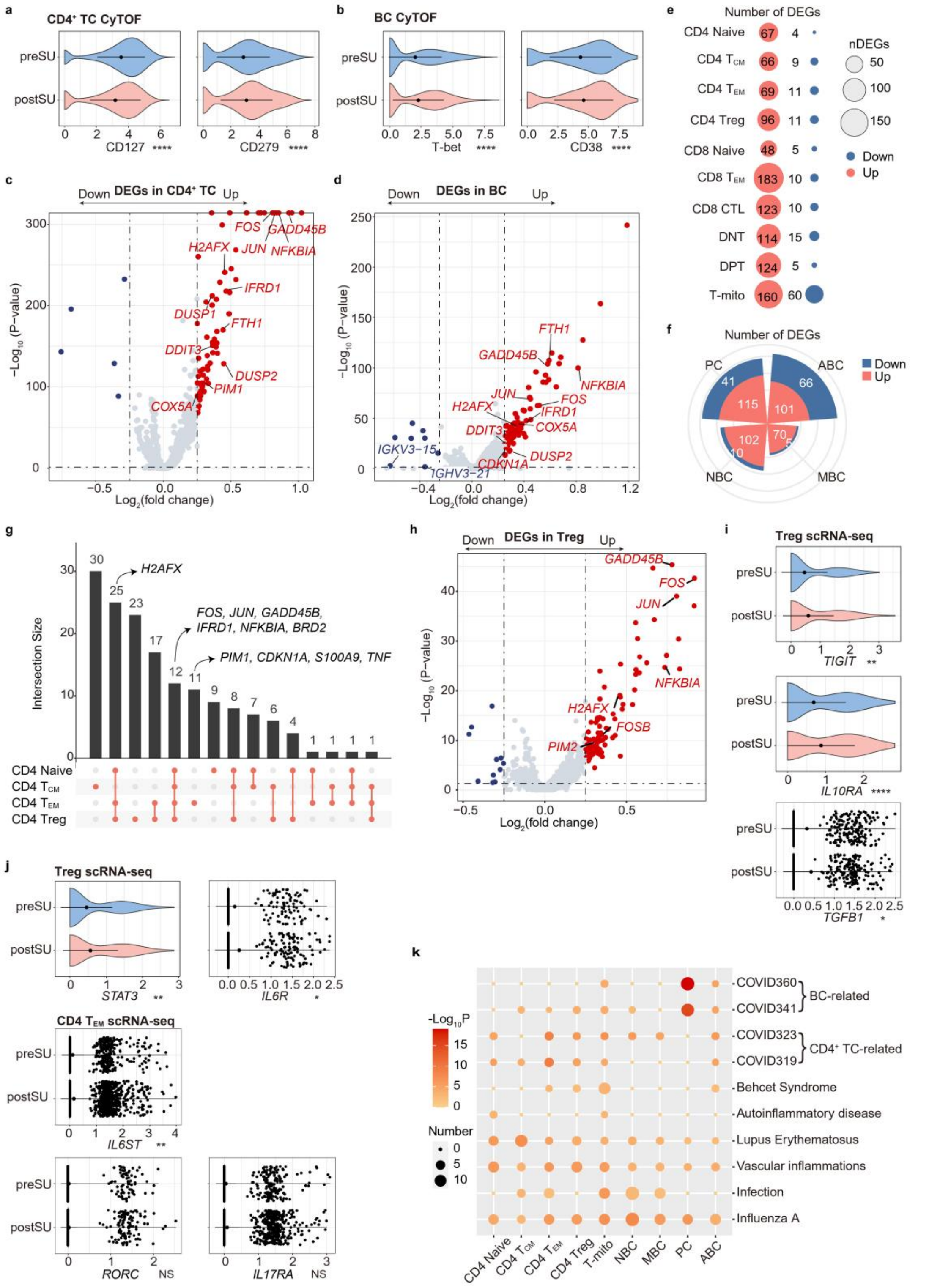

Supplementary Figure 5. Changes in proteomic and transcriptional profiles of CD4⁺ TC, T-mito and BC.

a Violin plot showing the expression of CD127 and CD279 in CD4⁺ TC between preSU and postSU groups in CyTOF. **b** Violin plot showing the expression of T-bet and CD38 in BC between preSU and postSU groups in CyTOF. **c** Volcano plot showing DEGs in CD4⁺ TC between preSU and postSU groups. **d** Volcano plot showing DEGs in BC between preSU and

postSU groups. **e** Dot plot showing the numbers of DEGs of TC subsets. **f** Rose diagram showing the numbers of DEGs in BC subsets. **g** UpSet Plot showing the integrated comparative analysis of upregulated DEGs in $CD4^+$ TC subsets. **h** Volcano plot showing DEGs in Treg between preSU and postSU groups. **i** Violin plot showing the expression of *TIGIT*, *IL10RA*, *TGFB1* in Treg between preSU and postSU groups in scRNA-seq. **j** Violin plot showing the expression of *STAT3*, *IL6R* in Treg, and *IL6ST*, *RORC* and *IL17RA* in CD4 T_{EM} between preSU and postSU groups in scRNA-seq. **k** Representative COVID and DisGeNET terms in $CD4^+$ TC, T-mito and BC subsets upregulated by SU. For the box plot within each violin plot, middle lines indicate median values, boxes range from the 25th to 75th percentiles. Significance in a, b, i and j was calculated using two-sided Wilcoxon test as implemented in the function "compare_means" with default parameters; **P < 0.01, ****P < 0.0001, NS (not significant).

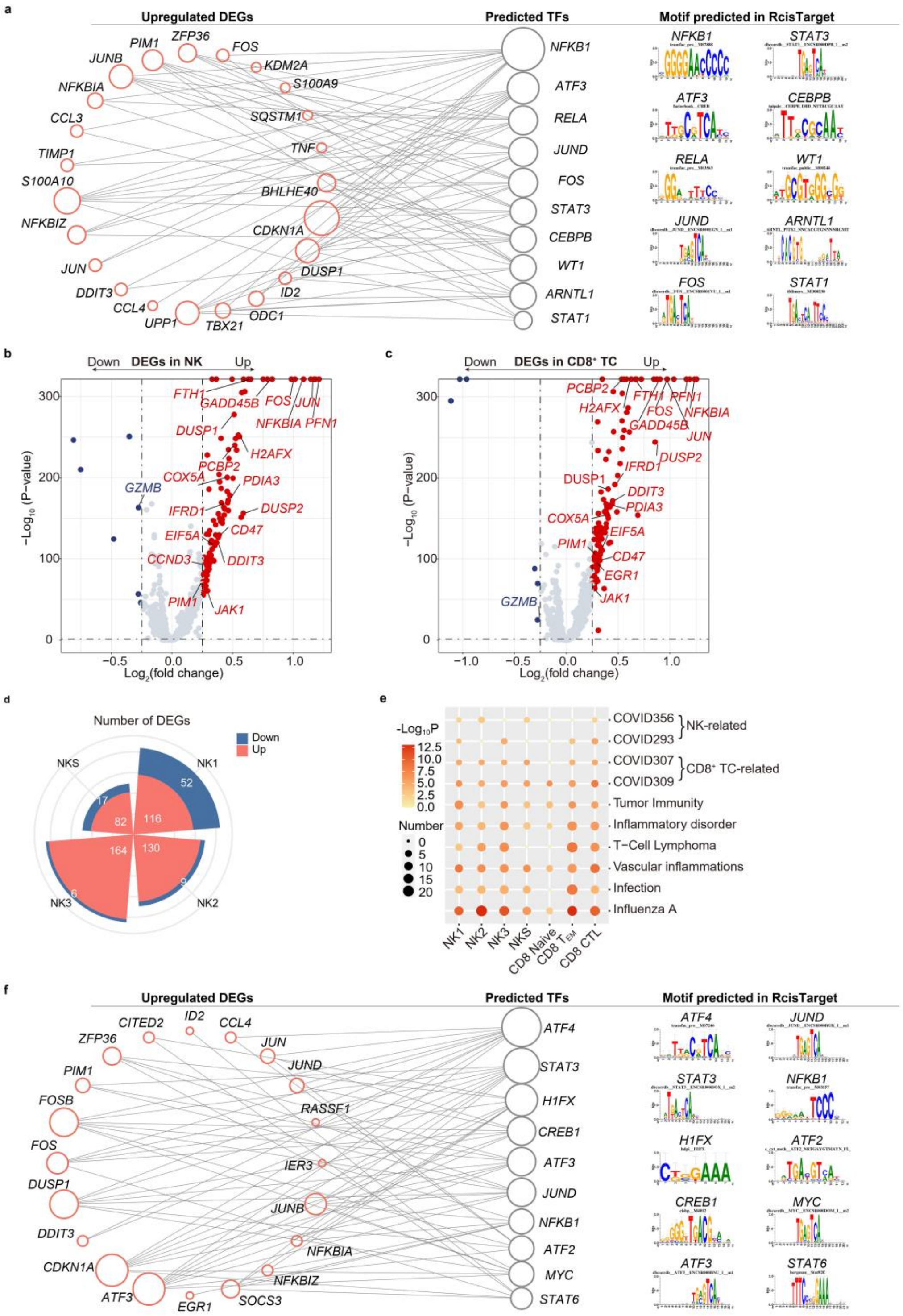

Supplementary Figure 6. Changes in transcriptional profiles of NK and CD8⁺ TC.

a Network visualization of the predicted transcriptional regulatory networks in CD4⁺ TC, T-mito and BC subsets enhanced by SU using RcisTarget tool. **b** Volcano plot showing DEGs in NK between preSU and postSU groups. **c** Volcano plot showing DEGs in CD8⁺ TC between preSU and postSU groups. **d** Rose diagram showing the numbers of DEGs in NK subsets. **e** Representative COVID and DisGeNET terms in NK and CD8⁺ TC subsets upregulated by SU. **f** Network visualization of the predicted transcriptional regulatory networks in NK and CD8⁺ TC subsets enhanced by SU using RcisTarget tool.

Figure. S7

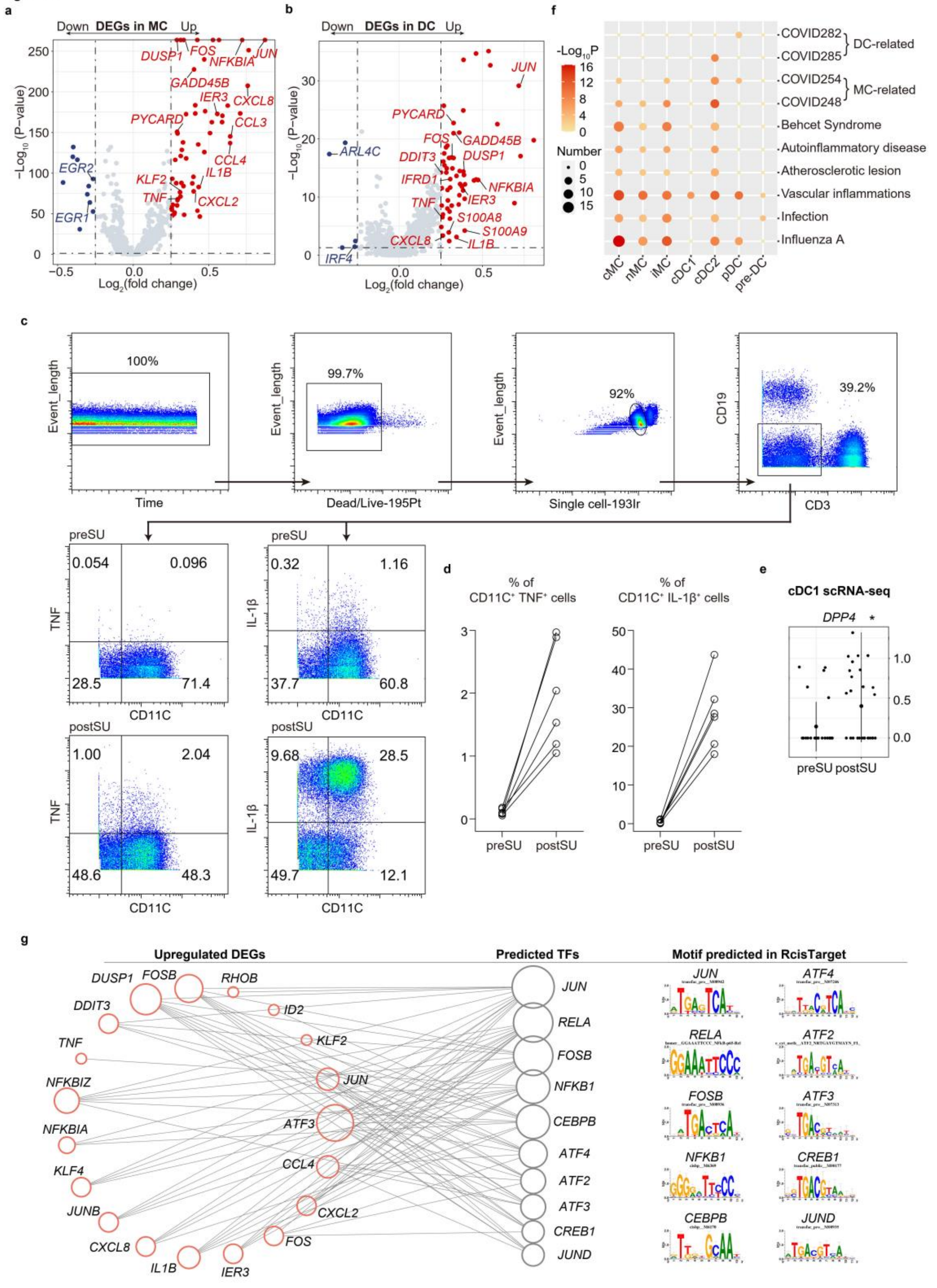

Supplementary Figure 7. Changes in transcriptional profiles of myeloid cell.

a Volcano plot showing DEGs in MC between preSU and postSU groups. **b** Volcano plot showing DEGs in DC between preSU and postSU groups. **c** Gating strategy for CD11C⁺ TNF⁺ cells and CD11C⁺ IL-1β⁺ cells using CyTOF. **d** The percentage of CD11C⁺ TNF⁺ cells and CD11C⁺ IL-1β⁺ cells in CD3⁻CD19⁻ cells between preSU and postSU groups (n = 6 /group). **e** Violin plot showing the expression of *DPP4* in cDC1 between preSU and postSU groups. **f**

Representative COVID and DisGeNET terms in myeloid cell subsets upregulated by SU. **g** Network visualization of the predicted transcriptional regulatory networks in myeloid cell subsets enhanced by SU using RcisTarget tool. For the box plot within each violin plot, middle lines indicate median values, boxes range from the 25th to 75th percentiles. Significance in d was calculated using two-tailed paired t test; significance in e was calculated using two-sided Wilcoxon test as implemented in the function “compare_means” with default parameters; $*P < 0.05$.

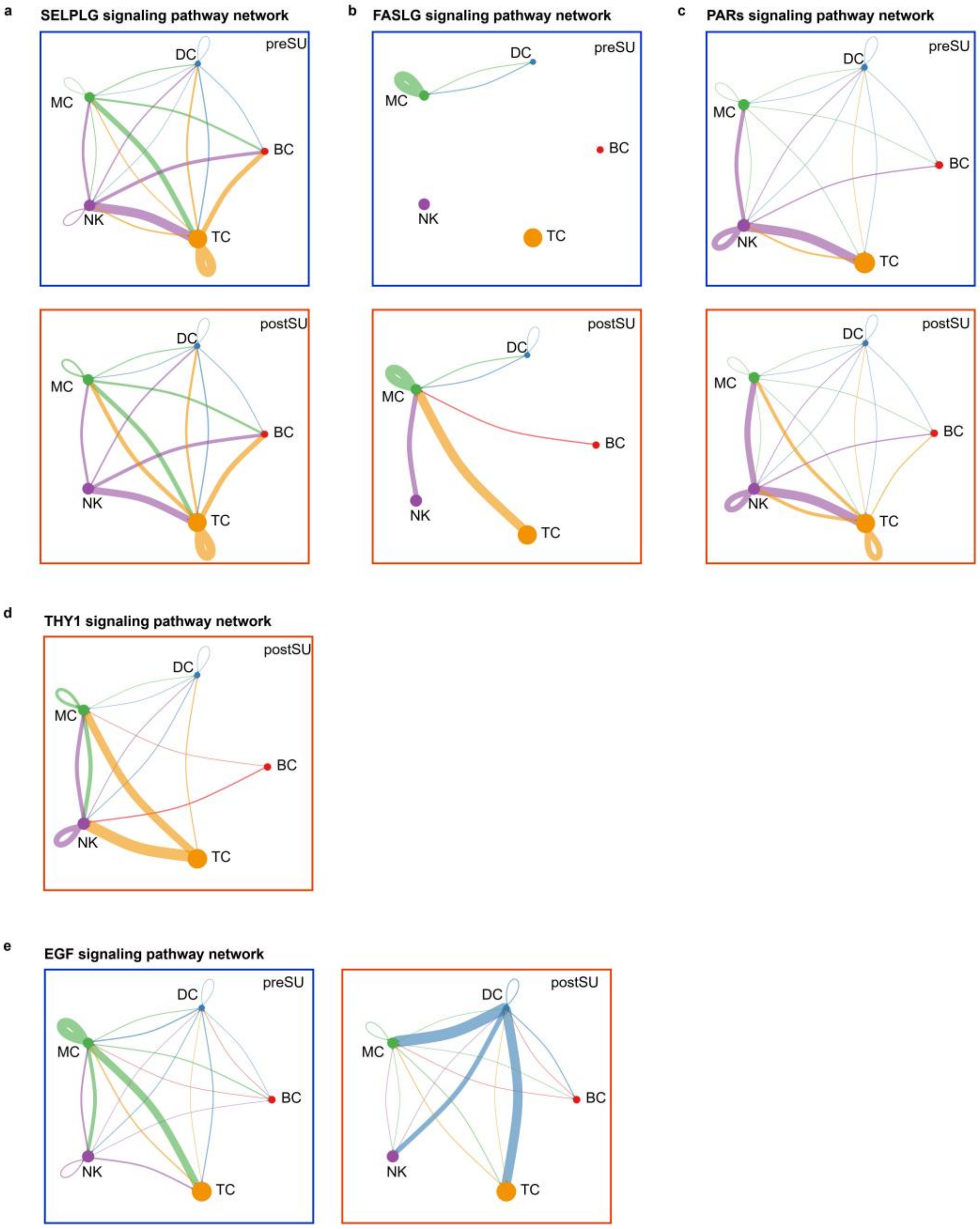

Supplementary Figure 8. Changes in the cellular communication.

a Circle plot showing the inferred SELPLG signaling networks. **b** Circle plot showing the inferred FASLG signaling networks. **c** Circle plot showing the inferred PARs signaling networks. **d** Circle plot showing the inferred THY1 signaling networks in postSU group. **e** Circle plot showing the inferred EGF signaling networks.

Supplementary Table

Supplementary Table 1. Subjects' information.

Ident	Group	Gender	Age	Disease	scRNA-seq	CyTOF panel	BMI, kg/m^2
preSU1	preSU	female	39	-	√	√	19.14
preSU2	preSU	male	50	-	√	√	20.83
preSU3	preSU	female	40	-	√	√	25.02
preSU4	preSU	male	51	-	√	√	21.19
preSU5	preSU	male	52	-	√	√	22.33
preSU6	preSU	female	52	-	√	√	23.98
postSU1	postSU	female	39	-	√	√	18.98
postSU2	postSU	male	50	-	√	√	20.9
postSU3	postSU	female	40	-	√	√	25.02
postSU4	postSU	male	51	-	√	√	21.26
postSU5	postSU	male	52	-	√	√	22.46
postSU6	postSU	female	52	-	√	√	24.14

Supplementary Table 2A. CyTOF antibodies.

Name	Conjugated Metal	Source	Catalogue Number	marker_class
CD11C	147Sm	Fluidigm	3147008B	type
CD56/NCAM1	176Yb	Fluidigm	3176008B	type
CD14	175Lu	Fluidigm	3175015B	type
CD279/PD-1	155Gd	Fluidigm	3155009B	type
CD196/CCR6	141Pr	Fluidigm	3141003A	type
CD3	154Sm	Fluidigm	3154003B	type
CD45	89Y	Fluidigm	3089003B	type
CD16	209Bi	Fluidigm	3209002B	type
CD25	150Nd	BD	555430	type
CD123	143Nd	BD	555642	type
CCR7	151Eu	&D Systen	MAB197-100	type
CD19	172Yb	BioLegend	302202	type
CD57	114Cd	BioLegend	359602	type
CD8A	111Cd	BioLegend	301002	type
CD4	116Cd	BioLegend	300502	type
CD27	158Gd	BD	555439	type
CD45RO	165Ho	BioLegend	304239	type
CLEC9A	161Dy	BioLegend	353802	type
CD45RA	153Eu	BioLegend	304143	type
HLA-DR	112Cd	BioLegend	307651	type
CD20	113Cd	BioLegend	302302	type
CD1C	166Er	BioLegend	331502	type
Foxp3	159Tb	Fluidigm	3159028A	type
CD152/CTLA4	170Er	Fluidigm	3170005B	type
KI67	168Er	BioLegend	350502	type
GZMB	171Yb	BioLegend	372202	type
GZMK	174Yb	BioLegend	370502	type
LEF1	142Nd	BioLegend	616002	type
MZB1	173Yb	hermoFish	PA5-80838	type
T-bet	160Gd	BioLegend	644825	type
CD102	146Nd	BioLegend	328502	type
CD105	162Dy	BioLegend	323202	type
CCR4	149Sm	Fluidigm	3149029A	state
CD183/CXCR3	163Dy	Fluidigm	3163004B	state
CD38	164Dy	BioLegend	303502	state
CD127	148Nd	BioLegend	351302	state
CCL5	145Nd	Novus	MAB278-100	state
GATA3	167Er	Novus	MAB6330	state

Supplementary Table 2B. Cell clustering strategy in CyTOF.

**Celltypes**		Markers Positive	Negative
T cell	CD4 Naive	CD4 CD45RA $CCR7^{high}$	CD45RO
	CD4 T_{CM}	CD4 CD45RO $CCR7^{high}$	
	CD4 T_{EM}	CD4 CD45RO $CCR7^{low}$	CD45RA
	CD4 Treg	CD4 CD25	
	CD4 CTL	CD4 CD57	
	CD4 Tex	CD4 CD279	
	CD8 Naive	CD8 CD45RA $CCR7^{high}$	CD45RO
	CD8 T_{EM}	CD8 CD45RO	CD45RA
	CD8 CTL	CD8 CD57	
	CD8 Tex	CD8 CD279	
	DNT		CD4 CD8
	DPT	CD4 CD8	
	T-mito	KI67	
Natural Killer cell	NK1	$CD16^{low}$ $CD56^{bright}$	
	NK2	$CD16^{high}$ $CD56^{dim}$	CD57
	NK3	$CD16^{high}$ $CD56^{dim}$ $CD57^{+}$	
B cell	NBC	CD19 CD20 IGHD	
	MBC	CD19 CD27	
	PC	CD19 CD38	CD20
	ABC	CD19 CD11C	
Monocyte	cMC	$CD14^{high}$	CD16
	nMC	$CD14^{+}CD16^{+/-}$	
	iMC	$CD14^{+/-}CD16^{high}$	
Dendritic Cell	cDC	CD11C HLA-DR	CD123
	pDC	CD123 CD38	HLA-DR

Supplementary Table 3. Cell clustering strategy in scRNA-seq.

**Celltypes**		Markers	
		Positive	Negative
CD34		*CD34 SOX4*	
Megakaryocyte		*PF4 PPBP TUBB1*	
T cell	CD4 Naive	*CD4 CCR7*^{high} *CD69*^{low}	
	CD4 T_{CM}	*CD4 CCR7*^{med} *CD69*^{high} or *AQP3*^{high}	
	CD4 T_{EM}	*CD4 CCR6*	*CCR7*
	CD4 Treg	*CD4 FOXP3*	
	CD8 Naive	*CD8 CCR7 LEF1*	
	CD8 T_{EM}	*CD8 GZMK*	
	CD8 CTL	*CD8 GNLY*	
	DNT		*CD4 CD8*
	DPT	*CD4 CD8*	
	T-mito	*STMN1 MKI67*	
Natural Killer cell	NK1	*FCGR3A*^{low} *NCAM1*^{bright}	
	NK2	*FCGR3A*^{high} *NCAM1*^{dim}	*B3GAT1*
	NK3	*FCGR3A*^{high} *NCAM1*^{dim} *B3GAT1*$^{+}$	
	NKS	*S100B*$^{+}$	
B cell	NBC	*CD19 IL4R IGHD*	
	MBC	*CD19 CD27 IGHG1*	
	PC	*CD19 MZB1*	
	ABC	*CD19 ITGAX*	
Monocyte	cMC	*CD14*^{high}	*FCGR3A*
	nMC	*CD14*$^{+}$*FCGR3A*$^{+/-}$	
	iMC	*CD14*$^{+/-}$*FCGR3A*^{high}	
Dendritic Cell	cDC1	*CLEC9A THBD*	
	cDC2	*CD1C*	
	pDC	*CLEC4C IL3RA*	*AXL*
	pre-DC	*AXL IL3RA*	
