# Supplementary material for: Effects of poor sleep on the immune cell landscape as assessed by single-cell analysis
Source: Commun Biol. 2021 Nov 25;4:1325. doi: 10.1038/s42003-021-02859-8 (PMC8617259; doi:10.1038/s42003-021-02859-8)
Supplement: Supplementary file 2 — Description of Additional Supplementary Files [file 42003_2021_2859_MOESM2_ESM.pdf]

# Description of Additional Supplementary Files

**File name:** Supplementary Data 1.
**Description:** The detailed DEGs dataset in five major immune cells.

**File name:** Supplementary Data 2.
**Description:** The detailed DEGs dataset in TC subsets.

**File name:** Supplementary Data 3.
**Description:** The detailed DEGs dataset in NK subsets.

**File name:** Supplementary Data 4.
**Description:** The detailed DEGs dataset in BC subsets.

**File name:** Supplementary Data 5.
**Description:** The detailed DEGs dataset in MC subsets.

**File name:** Supplementary Data 6.
**Description:** The detailed DEGs dataset in DC subsets.

**File name:** Supplementary Data 7.
**Description:** The functional signature with the full gene list used in calculating scores of biological processes.

**File name:** Supplementary Data 8.
**Description:** The detailed cell counts in CyTOF.

**File name:** Supplementary Data 9.
**Description:** Source data underlying plots shown in main figures.
