# Supplementary material for: Effects of poor sleep on the immune cell landscape as assessed by single-cell analysis
Source: Commun Biol. 2021 Nov 25;4:1325. doi: 10.1038/s42003-021-02859-8 (PMC8617259; doi:10.1038/s42003-021-02859-8)
Supplement: Supplementary file 12 — Reporting Summary [file 42003_2021_2859_MOESM12_ESM.pdf]

nature portfolio

Corresponding author(s): Wenru Su, Yingfeng Zheng

Last updated by author(s): Oct 16, 2021

Reporting Summary

Nature Portfolio wishes to improve the reproducibility of the work that we publish. This form provides structure for consistency and transparency in reporting. For further information on Nature Portfolio policies, see our Editorial Policies and the Editorial Policy Checklist.

Statistics

For all statistical analyses, confirm that the following items are present in the figure legend, table legend, main text, or Methods section.

n/a	Confirmed	
☐	☒	The exact sample size (*n*) for each experimental group/condition, given as a discrete number and unit of measurement
☐	☒	A statement on whether measurements were taken from distinct samples or whether the same sample was measured repeatedly
☐	☒	The statistical test(s) used AND whether they are one- or two-sided<br>*Only common tests should be described solely by name; describe more complex techniques in the Methods section.*
☒	☐	A description of all covariates tested
☐	☒	A description of any assumptions or corrections, such as tests of normality and adjustment for multiple comparisons
☐	☒	A full description of the statistical parameters including central tendency (e.g. means) or other basic estimates (e.g. regression coefficient) AND variation (e.g. standard deviation) or associated estimates of uncertainty (e.g. confidence intervals)
☒	☐	For null hypothesis testing, the test statistic (e.g. *F*, *t*, *r*) with confidence intervals, effect sizes, degrees of freedom and *P* value noted<br>*Give P values as exact values whenever suitable.*
☒	☐	For Bayesian analysis, information on the choice of priors and Markov chain Monte Carlo settings
☒	☐	For hierarchical and complex designs, identification of the appropriate level for tests and full reporting of outcomes
☒	☐	Estimates of effect sizes (e.g. Cohen's *d*, Pearson's *r*), indicating how they were calculated

Our web collection on statistics for biologists contains articles on many of the points above.

Software and code

Policy information about availability of computer code

Data collection	In scRNA-seq, single-cell suspensions were converted to barcoded scRNA-seq libraries using the Chromium Single Cell 5′ Library (10X Genomics, Genomics chromium platform Illumina NovaSeq 6000), Gel Bead and Multiplex Kit, and Chip Kit (10X Genomics). The Chromium Single Cell 5′ v2 Reagent Kit (120237; 10X Genomics) was used to prepare single-cell RNA libraries according to the manufacturer's instructions. FastQC software was used to check library quality.<br>In CyTOF, after live cell barcoding, surface staining and intracellular factor staining, CyTOF data were obtained from a SuperSampler fluidics CyTOF2 system (Victorian Airships, Alamo, CA), at an event rate of < 400 /s, and then normalized with Helios normalizer software (version 6.7.1016; Fluidigm). Quality control and tuning of the CyTOF2 mass cytometer (Fluidigm) was performed daily. Cytobank software (version 7.0; https://mtsinai.cytobank.org) was used to deconvolute barcoded samples and filter cross-sample doublets.
Data analysis	CellRanger v4.0 was used to aline the sequencing reads to human transcriptome. Further data processing was performed by CellRanger v4.0 and Seurat package v3.0. The Metascape webtool (www.metascape.org) was used to conduct the studies of GO biological process and pathways, DisGeNET, COVID, and TRRUST, allowing us to visualize the functional patterns of gene clusters and conduct statistical analysis. Cell-cell communication was predicted by iTALK (https://github.com/Coolgenome/iTALK) and CellChat (https://github.com/sqjin/CellChat) R packages. RcisTarget package v1.8.0 was used to perform motif-enrichment to predict upstream regulators.<br>All cytometry data were transformed with an inverse hyperbolic sine (arcsinh) function (mass cytometry: cofactor of 5) using R. FlowCore was used to read and process the CyTOF data. Further CyTOF analysis was performed by R package CATALYST and diffcyt.<br>GraphPad Prism v8.0.2 was used for data analysis and presentation.

For manuscripts utilizing custom algorithms or software that are central to the research but not yet described in published literature, software must be made available to editors and reviewers. We strongly encourage code deposition in a community repository (e.g. GitHub). See the Nature Portfolio guidelines for submitting code & software for further information.

Data

Policy information about availability of data

All manuscripts must include a data availability statement. This statement should provide the following information, where applicable:

- Accession codes, unique identifiers, or web links for publicly available datasets
- A description of any restrictions on data availability
- For clinical datasets or third party data, please ensure that the statement adheres to our policy

The data that support the findings of this study are available from the corresponding author upon request. The scRNA-seq data is deposited in the Genome Sequence Archive in BIG Data Center, Beijing Institute of Genomics (BIG, https://bigd.big.ac.cn/gsa-human/), Chinese Academy of Sciences, under the Project Accession No. PRJCA004314 and GSA Accession No. HRA000604. Experimental protocols and the data analysis pipeline used in our work follow those described on the 10X Genomics and Seurat official websites. The analysis steps, functions, and parameters used are described in detail in the Methods section.

Field-specific reporting

Please select the one below that is the best fit for your research. If you are not sure, read the appropriate sections before making your selection.

☒ Life sciences ☐ Behavioural & social sciences ☐ Ecological, evolutionary & environmental sciences

For a reference copy of the document with all sections, see nature.com/documents/nr-reporting-summary-flat.pdf

Life sciences study design

All studies must disclose on these points even when the disclosure is negative.

Sample size	The 12 human blood samples were collected from 6 individual healthy donors, respectively pre- and post-24h-sleep loss. Sample size was determind based on the knowledge on appropriate sample size to ensure adequete data for reliable assessment.
Data exclusions	We applied the following filtering parameters: (i) all genes that were not detected in ≥3 cells were discarded; (ii) cells with less than 200 total unique transcripts were removed prior to downstrem analysis, and (iii) celss in which > 15% of the UMIs mapped to the mitochondrial genes were filtered.
Replication	We included as many healthy controls as possible, the analysis showed a good correlation. Data analysis was performed by more than two separate investigators.
Randomization	No randomization was used in this study.
Blinding	Blinding is not possible as the experimental design was a pre-post study in the same patient.

Reporting for specific materials, systems and methods

We require information from authors about some types of materials, experimental systems and methods used in many studies. Here, indicate whether each material, system or method listed is relevant to your study. If you are not sure if a list item applies to your research, read the appropriate section before selecting a response.

Materials & experimental systems

n/a	Involved in the study	
☒	☐	Antibodies
☒	☐	Eukaryotic cell lines
☒	☐	Palaeontology and archaeology
☒	☐	Animals and other organisms
☐	☒	Human research participants
☒	☐	Clinical data
☒	☐	Dual use research of concern

Methods

n/a	Involved in the study	
☒	☐	ChIP-seq
☒	☐	Flow cytometry
☒	☐	MRI-based neuroimaging

Human research participants

Policy information about studies involving human research participants

Population characteristics	Six healthy participants (aged 39-52, BMI 19-25, 3 males and 3 females) were recruited for the study. Distribution of gender, age was summarized in Supplementary Table 1.
Recruitment	The subjects were recruited through voluntary enrollment. To be eligible for study participation, subjects met the following

Recruitment	inclusionary criteria: age range from 35 to 55 y; physical and psychological health; no clinically significant abnormalities in blood chemistry; regular sleep habits and a steady sleep time of approximately 8 h (22.00–06.00 h). Exclusion criteria included any physiological or psychiatric pathology, medication, smoking, obesity, binge drinking, or excessive caffeine use (> 3 cups per day), extreme morningness, extreme eveningness, sleep or circadian disorders.
Ethics oversight	All study participants provided informed consent, and the study design was approved by the appropriate ethics review board.

Note that full information on the approval of the study protocol must also be provided in the manuscript.
